# Analysis of ductal carcinoma in situ by self-reported race reveals molecular differences related to outcome

**DOI:** 10.1186/s13058-024-01885-8

**Published:** 2024-09-02

**Authors:** Siri H. Strand, Kathleen E. Houlahan, Vernal Branch, Lorraine M. King, Thomas Lynch, Belén Rivero-Guitiérrez, Bryan Harmon, Fergus Couch, Kristalyn Gallagher, Mark Kilgore, Shi Wei, Angela DeMichele, Tari King, Priscilla McAuliffe, Christina Curtis, Kouros Owzar, Jeffrey R. Marks, Graham A. Colditz, E. Shelley Hwang, Robert B. West

**Affiliations:** 1 Department of Pathology, Stanford University School of Medicine, Stanford, CA 94305 USA; 2 Stanford Cancer Institute, Stanford University School of Medicine, Stanford, CA 94305 USA; 3 Department of Medicine, Genetics, Biomedical Data Science, Stanford University School of Medicine, Stanford, CA 94305 USA; 4 National Breast Cancer Coalition, 2001 L Street NW, Suite 500 PMB#50111, Washington, DC 20036 USA; 5 Department of Surgery, Duke University School of Medicine, Durham, NC 27708 USA; 6 Department of Pathology, Montefiore Medical Center, New York City, NY USA; 7 Department of Pathology, Mayo Clinic, Rochester, MN USA; 8 Department of Surgery, University of North Carolina at Chapel Hill, Chapel Hill, NC USA; 9 Department of Pathology, University of Washington, Seattle, WA USA; 10 Department of Pathology, University of Alabama at Birmingham, Birmingham, AL USA; 11 Department of Medicine, University of Pennsylvania, Philadelphia, PA USA; 12 Department of Surgery, Brigham and Women’s Hospital, Boston, MA USA; 13 Department of Surgery, University of Pittsburgh, Pittsburgh, PA USA; 14 Duke Cancer Institute, Duke University School of Medicine, Durham, NC 27708 USA; 15 Department of Biostatistics & Bioinformatics, Duke University School of Medicine, Durham, NC 27708 USA; 16 Department of Surgery, Washington University School of Medicine, St. Louis, MO 63110 USA

**Keywords:** Ductal carcinoma in situ, Breast cancer, Outcome, Recurrence, Progression, Race, Ancestry

## Abstract

**Background:**

Ductal carcinoma in situ (DCIS) is a non-obligate precursor to invasive breast cancer (IBC). Studies have indicated differences in DCIS outcome based on race or ethnicity, but molecular differences have not been investigated.

**Methods:**

We examined the molecular profile of DCIS by self-reported race (SRR) and outcome groups in Black (*n* = 99) and White (*n* = 191) women in a large DCIS case-control cohort study with longitudinal follow up.

**Results:**

Gene expression and pathway analyses suggested that different genes and pathways are involved in diagnosis and ipsilateral breast outcome (DCIS or IBC) after DCIS treatment in White versus Black women. We identified differences in ER and HER2 expression, tumor microenvironment composition, and copy number variations by SRR and outcome groups.

**Conclusions:**

Our results suggest that different molecular mechanisms drive initiation and subsequent ipsilateral breast events in Black versus White women.

**Supplementary Information:**

The online version contains supplementary material available at 10.1186/s13058-024-01885-8.

## Background

Ductal carcinoma in situ (DCIS) is a preinvasive neoplastic lesion of the breast that reflects increased risk for invasive breast cancer (IBC) [1]. DCIS encompasses a heterogenous group of lesions, the natural history of which is not well-understood. The incidence of DCIS in the U.S. has increased greatly over the past 40 years, mainly due to more widespread screening mammography, which is the most common mode of DCIS detection [2]. Treatment for DCIS includes mastectomy or breast-conserving surgery with or without radiation or endocrine therapy. However, research indicates many women are over-diagnosed and over-treated, and trials on active surveillance cohorts are ongoing to better understand the factors that lead to DCIS disease progression [3–5].

Several studies have demonstrated a significant variation in risk of developing IBC after DCIS by race or ethnicity. Overall, results indicate that Black women are more likely to have recurring invasive and noninvasive tumors in either breast, as well as overall higher IBC mortality rates after DCIS, compared to White women [6–12]. In these studies, racial or ethnic risk disparities could not be attributed to histologic features or treatment of DCIS [10, 12] though subsequent IBC after DCIS was biologically more aggressive among Black than White women [11].

While race and ethnicity are social constructs, ancestry refers to a person’s genetic admixture reflecting ancestral lineage [13]. Understanding the variation in response explained by self-reported race is critically important in the context of cancer health disparities research as it may reveal information orthogonal to that provided by genetic ancestry [14]. Studies have revealed molecular differences by race, ethnicity, or ancestry in invasive cancers, including IBC [15–18], but these have not yet been fully investigated in DCIS or other precancers, due to marked underrepresentation of minority populations in most cohorts. We recently generated the Human Tumor Atlas Network (HTAN) DCIS Atlas [19, 20], where we identified molecular changes in DCIS at the DNA, RNA and protein levels. This work showed that many of the molecular alterations found in IBC are already present at the earlier DCIS stage, implying that any racial differences in IBC may arise at this precursor stage. In the present study, we leveraged the large amount of metadata available for two HTAN DCIS cohorts to analyze molecular features and DCIS outcome by self-reported race (SRR) and inferred global ancestry from DNA sequencing data to facilitate analysis by both ancestry and SRR.

## Methods

### Patient samples

We used data from two previously described case-control cohorts of patients diagnosed with pure DCIS with or without a subsequent ipsilateral breast event (iBE, either DCIS or IBC) after surgical treatment [19]. Both cohorts included women self-described as White (n_TBCRC_ = 128, n_RAHBT_ = 63), and Black (n_TBCRC_ = 65, n_RAHBT_ = 34). The cohorts were combined to improve statistical power to allow comparison by SRR. Identical eligibility criteria were used for outcome analysis in both cohorts. Briefly, The Resource of Archival Breast Tissue (RAHBT) cohort includes women ≥ 18 years of age with documented cases of premalignant breast disease including DCIS. TBCRC 038 is a retrospective cohort with patients from 12 participating TBCRC (Translational Breast Cancer Research Consortium) sites, which includes women treated for DCIS at one of the enrolling institutions between 01/01/1998 and 02/29/2016.

For the combined cohort studied here, samples from the index (primary) lesion from a total of 313 patients (n_TBCRC_ = 216, n_RAHBT_ = 97) were previously analyzed by RNA-seq. We excluded 17 patients with missing SRR, as well as patients with SRR listed as Asian (*n* = 5) and Pacific Islander (*n* = 1) due to low numbers in these groups. The final cohort consisted of 290 patients (Table 1) and was composed of 34.1% Black women and 65.9% White women. For White women, the median age at diagnosis was 51.6 years, and median year of diagnosis was 2007. Median time to recurrence with ipsilateral IBC was 60 months, and time to diagnosis of ipsilateral DCIS was 40.8 months. For White women with no iBEs, median follow-up extended to 128.5 months. Treatment of initial DCIS ranged from lumpectomy with radiation (55.0%), and no radiation (12.6%) to mastectomy (31.9%). For Black women, the median age at diagnosis was 54 years, and median year of diagnosis was 2009. Median time to recurrence with ipsilateral IBC was 47 months, and time to diagnosis of ipsilateral DCIS was 35.5 months. For Black women with no iBEs, median follow-up was 91.5 months. There was no significant difference in right censorship rates between White (*n* = 4) and Black (*n* = 1) women at 5 years (*P* = 1.0). Treatment of initial DCIS ranged from lumpectomy with radiation (67.7%), and no radiation (14.1%) to mastectomy (15.2%). Tumor low-pass WGS data was available for 208 patients (71.7%), 33.7% of whom were Black.

**Table 1:** Combined breast pre-cancer Atlas patient cohorts stratified by SRR

	Black			White		
	Controls	Cases		Controls	Cases	
	DCIS without recurrence	DCIS with DCIS Recurrence	DCIS with Invasive Recurrence	DCIS without recurrence	DCIS with DCIS Recurrence	DCIS with Invasive Recurrence
	**(** *n* ** = 46)**	**(** *n* ** = 26)**	**(** *n* ** = 27)**	**(** ***n*** ** = 106)**	**(** ***n*** ** = 48)**	**(** ***n*** ** = 37)**
**Year of Diagnosis**						
Median	2011	2008	2007	2007	2007	2005
**Age at Diagnosis**						
Median	53.9	58	49	51.8	51.3	52
Mean (± SD)	54.6 (8.7)	56.8 (8.1)	52.5 (10.3)	53.6 (7.7)	52.9 (9.2)	53.0 (9.3)
**Grade**						
1	8	7	1	13	2	4
2	22	8	14	39	19	11
3	16	11	12	54	27	22
**DCIS size (cm)**						
Median	1.7	2.0	1.8	2.5	1.3	2.0
Mean (± SD)	2.2 (1.6)	2.6 (2.4)	2.4 (2.0)	2.9 (2.0)	2.1 (1.9)	2.9 (2.3)
**Marker Status**						
ER(-)	11	6	9	35	25	10
ER(+)	35	20	18	71	23	27
HER2(-)	37	22	21	75	22	29
HER2(+)	9	4	6	31	26	8
**Treatment**						
Lumpectomy -Radiation	4	7	3	6	9	9
Lumpectomy + Radiation	35	17	15	61	29	15
Lumpectomy Radiation Unknown	0	1	2	1	0	0
Mastectomy	7	1	7	38	10	13
Time to Recurrence* (months)						
Median	91.5	35.5	47	128.5	40.8	60
Mean (± SD)	104.6 (39.3)	54.6 (45.1)	65.1 (45.2)	131.0 (48.3)	51.7 (37.8)	77.1 (52.6)
**Ancestry estimates**						
African						
Median	0.83	0.79	0.86	0.02	0.02	0.02
Mean (± SD)	0.79 (0.13)	0.73 (0.19)	0.80 (0.20)	0.06 (0.12)	0.04 (0.07)	0.05 (0.15)
European						
Median	0.14	0.20	0.12	0.96	0.97	0.97
Mean (± SD)	0.19 (0.13)	0.25 (0.18)	0.17 (0.18)	0.91 (0.13)	0.94 (0.09)	0.93 (0.15)
**Oncotype DCIS score**						
Median	9.93	9.95	10.15	10.08	10.21	10.06
Mean (± SD)	9.89 (0.45)	9.85 (0.56)	10.08 (0.41)	9.86 (0.74)	9.95 (0.96)	9.81 (0.88)

* To end of follow-up for no recurrence

### Sequencing and Multiplex Ion Beam Imaging (MIBI) data

We analyzed publicly available data including RNA and DNA sequencing, metadata, and MIBI data. We used copy number variation, Cibersort X, Oncotype DX DCIS scores, and PAM50 subtype calls from the HTAN DCIS atlas. For details see [19].

### Inference of global genetic ancestry

**Fig. 1:**
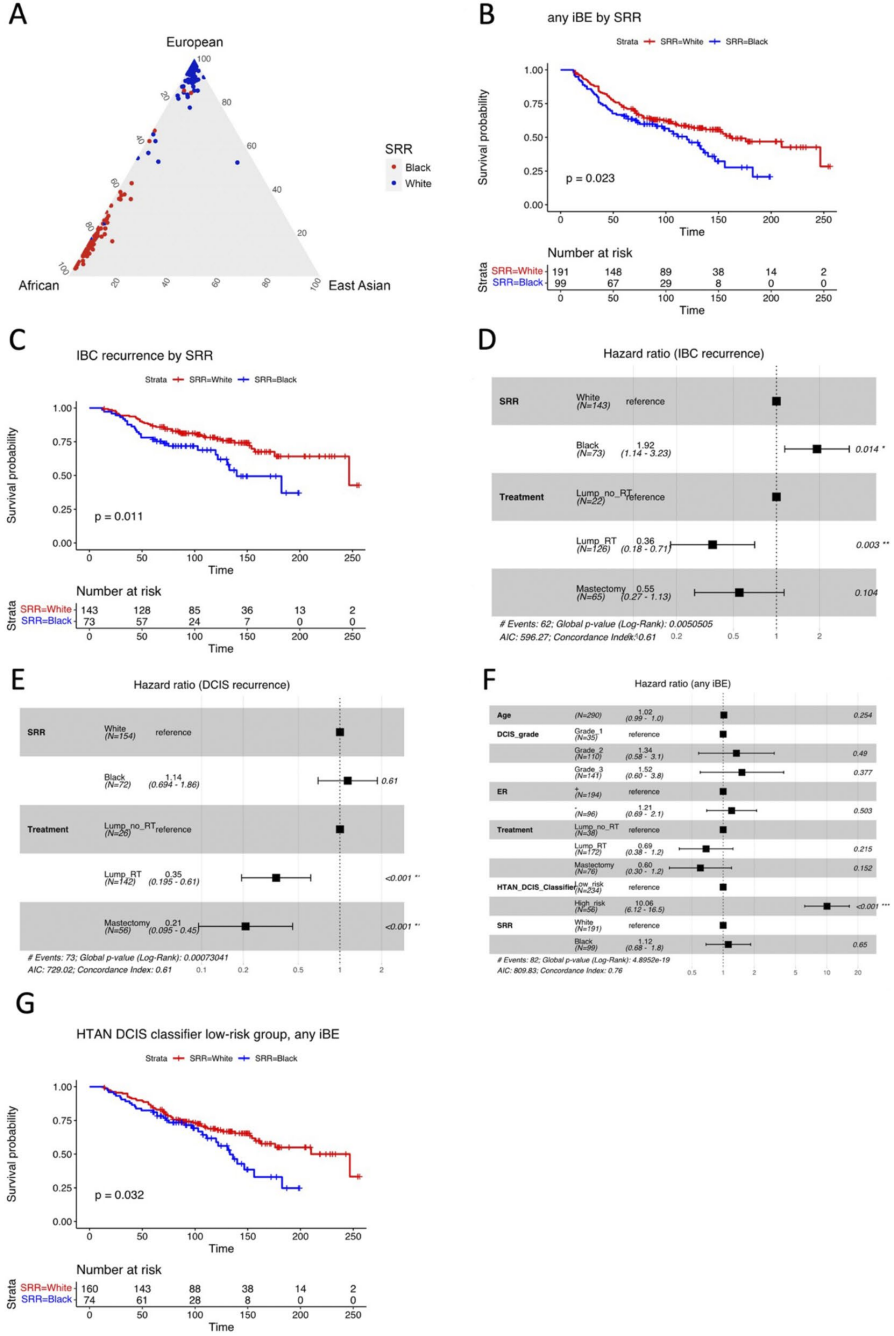
Outcome analysis by SRR. **A**) Ternary plot showing inferred ternary fractions of African, European, and East Asian ancestry for each of the 208 Women where DNA was available. Coloring indicates SRR. **B**) Kaplan-Meier plot of time to iBE (full follow-up) stratified by SRR. **C**) Kaplan-Meier plot of time to IBC recurrence only (full follow-up) stratified by SRR. **D**) Forest plot of multivariable Cox regression analysis including SRR and treatment type, for IBC recurrence only (full follow-up). **E**) Forest plot of multivariable Cox regression analysis including SRR and treatment type, for DCIS recurrence only (full follow-up). **F**) Forest plot of multivariable Cox regression analysis including the HTAN DCIS classifier, SRR, age at diagnosis, ER status, DCIS grade, and treatment, for any iBE (5-year outcome). **G**) Kaplan-Meier plot of time to iBE (full follow-up) in the HTAN DCIS classifier low-risk group stratified by SRR. **B**,** C**,** G**) P-values from log-rank tests

Given the low DNA sequence coverage of the WGS data, we leveraged QUILT (v0.1.9) [21] to genotype the TBCRC and RAHBT cohorts. QUILT is designed for genotyping low sequence coverage samples using a Gibbs sampling method to facilitate rapid imputation. Genotyping was conducted in batches considering 5 Mbp windows and 100 samples at a time. We used the 1000 Genomes Project as a reference panel (*n* = 3,202) [22]. We used default parameters apart from “–buffer 10000 –nGen 100”. The resulting variants were annotated with dbSNP (b155) and converted to EIGENSTRAT format using a combination of plink [23] (v1.9) and CONVERTF (v3.0). Finally, ancestry weights were inferred using SNPWEIGHTS [24] (v2.1) and the snpwt.CO reference panel. Ternary plot (Fig. 1A) was generated using the ggtern package (v. 3.4.2) in R (v. 4.2.2).

### Differential abundance analyses

Differential abundance analysis was performed using the R package DESeq2 v1.30.1 with default options. P-values were adjusted for multiple testing using the Benjamini-Hochberg method. False-discovery rate (FDR) < 0.05 was considered significant for all DESeq2 analyses.

### Gene set enrichment analyses

Gene set enrichment analyses were performed using the fgsea R package (v1.16.0) based on the MsigDB Hallmark, Kegg, Reactome, and Gene Ontology gene sets v2023.1. All genes from differential abundance analyses were included and were ranked by their signed adjusted P-values. Pathways with false-discovery rate (FDR) < 0.05 were considered significantly enriched.

Single sample gene set variation analysis was performed using the GSVA R package (v1.38.2) using default parameters.

### Outcome analysis

Associations with time to event were quantified using Cox Proportional Hazard model. Kaplan-Meier plots as implemented in the R packages survival (v3.3-1) and survminer (v0.4.9) were used to visualize outcome differences.

We previously generated the HTAN DCIS prognostic classifier [19] which was trained to predict iBEs within 5-years from treatment, regardless of treatment type. Time to recurrence analyses in the present paper were made either using 5-year follow-up, or full follow-up, as specified in the text.

### Statistical analyses

Wilcoxon rank-sum test was used to compare continuous distributions between two groups. Reported effect size was calculated as median(case)/median(control). Gene expression data.

were quantified as VST normalized reads generated using the DESeq2 R package (v1.30.1). All statistical analyses were implemented in the R statistical environment (v4.2.2). P-values were corrected for multiple hypothesis testing using the Benjamini-Hochberg method.

Association between categorical variables were analyzed and visualized using mosaic plots from the vcd (1.4–11) package in R with default Pearson’s chi-squared residuals. Boxplots, heatmaps, scatterplots and barplots were generated using the BoutrosLab.plotting.general R package v7.0.3 [25], or the R packages ggplot2 (v3.3.6, boxplots), or corrplot (v0.92, scatterplots).

## Results

### Clinical outcome analysis, stratified by SRR

Using the previously reported TBCRC and RAHBT cohorts, we collectively profiled the samples of primary DCIS lesions by SRR for 99 Black and 191 White women included in a case-control cohort (Table 1). Women diagnosed with pure DCIS with a subsequent ipsilateral breast event (iBE, either DCIS or IBC) after surgical treatment were designated as cases. Women diagnosed with pure DCIS without any subsequent iBE during time of follow-up were designated as **controls**. There was no difference in age at diagnosis between Black and White women, nor between cases and controls by SRR (Supplementary Figure S1 A-B).

While we previously analyzed these cohorts separately [19], we here combined them to enhance statistical power and thus allow analysis by SRR. To investigate the correlation between SRR and genomic ancestry in our dataset, we inferred global ancestry using whole genome sequencing (WGS) data from the combined cohort and compared these to SRR for the 208 patients where both parameters were available.

Similar to previous reports [26], we found that African ancestry was highly concordant with Black SRR, and European ancestry with White SRR (Fig. 1A). Due to the high level of agreement between genomic ancestry and SRR, and because WGS data and thus ancestry was not available for the entire study population, the following analyses described below were conducted using SRR to designate race.

To investigate outcome by SRR in the combined cohort, we first performed Kaplan-Meier analysis for time to any subsequent ipsilateral breast event (iBE), either DCIS or IBC recurrence. Here, Black women had significantly higher risk and shorter time to subsequent iBEs compared to White women (HR (95% CI): 1.5 (1.1–2.1), *P* = 0.023, Fig. 1B). Moreover, Black women had significantly shorter time and almost twice the risk of IBC recurrence specifically compared to White women (HR (95% CI): 1.9 (1.2–3.2), *P* = 0.011, Fig. 1C). This is in concordance with previous reports [6, 8, 9, 11, 12]. Conversely, no significant difference was observed for time to DCIS recurrence by SRR (HR (95% CI): 1.3 (0.83–2.2), *P* = 0.23, Supplementary Figure S1 C).

Next, we investigated the impact of different treatment modalities on outcome. We found a higher proportion of patients with recurrence in the lumpectomy only group compared with the lumpectomy + RT or mastectomy groups (*P* = 0.0007, Supplementary Figure S1 D). To investigate if treatment differences could explain the observed outcome disparity by SRR, we performed multivariable regression analysis including SRR and treatment using full follow-up. While treatment type was highly significantly associated with outcome (Lump + RT HR (95% CI): 0.44 (0.28–0.68), *P* < 0.001; Mastectomy HR (95% CI): 0.43 (0.26–0.73), *P* = 0.002, Supplementary Figure S1E), Black women had significantly higher risk of iBE after adjusting for treatment (HR (95% CI): 1.44 (1.01–2.06), *P* = 0.044, Supplementary Figure S1E). Moreover, Black women had 1.9 times higher risk of IBC recurrence compared to White women after adjusting for treatment (HR (95% CI): 1.92 (1.14–3.23), *P* = 0.014, Fig. 1D). Again, we observed no difference by SRR for DCIS recurrence only after adjusting for treatment (HR (95% CI): 1.14 (0.694–1.86), *P* = 0.61, Fig. 1E). We observed no significant difference in time to any iBE or IBC only by treatment (Supplementary Figure S1F-K), probably due to small sample sets for each treatment type. We previously calculated Oncotype DX DCIS score [27] from RNA-seq data for the entire study population included here [19]. While we observed no significant difference by SRR, we found that for both Black and White women, cases had significantly higher scores than controls (Black cases versus Black controls: *P* = 0.012. White cases versus White controls: *P* = 0.0092, Supplementary Figure S1L-M).

We recently presented the HTAN DCIS classifier consisting of 812 genes [19], which was trained to predict iBEs within 5 years from treatment in the TBCRC cohort, and then validated in the RAHBT cohort. To see how the classifier performed by SRR, we performed multivariable analysis including the predicted risk groups from the HTAN DCIS classifier, clinical variables (Age, DCIS grade, RNA-based ER-status, and treatment) and SRR for 5-year follow-up (Fig. 1F). We found that the HTAN DCIS classifier was highly predictive (Classifier high-risk group HR (95% CI): 10.06 (6.12–16.5), *P* < 0.001), without substantial contribution by SRR (Black HR (95% CI): 1.12 (0.68–1.8), *P* = 0.65). While the classifier performed well irrespective of SRR, we noted a trend towards enrichment of high-risk cases amongst Black women (*P* = 0.065, Supplementary Figure S1N). To further investigate the classifier’s performance by SRR, we performed Kaplan-Meier analysis of the Classifier High-risk and Low-risk groups, respectively, stratified by SRR. While no significant difference by SRR was observed in the High-risk group (*P* = 0.46, Supplementary Figure S1O, Black women classified as Low-risk had significantly shorter time to recurrence compared to White women (HR (95% CI) for Black women: 1.6 (1–2.5), *P* = 0.032, Fig. 1G). We noted that the biggest outcome difference was for late recurrences > 10 years from treatment, whereas the classifier was trained to predict recurrence ≤ 5 years from treatment.

Taken together, these results indicate there are differences between races in DCIS clinical outcome, with Black women having significantly shorter time to subsequent iBE, specifically IBC recurrence. Moreover, the HTAN DCIS classifier was highly informative in predicting iBE within 5 years from treatment for both Black and White women.

### Gene expression differences stratified by SRR and outcome

To investigate possible molecular differences underlying the observed DCIS outcome disparities between races, we performed differential gene expression analysis between primary DCIS from Black and White women. This analysis identified 384 differentially expressed (DE) genes (FDR < 0.05, Supplementary Table S1). Next, we performed gene set enrichment analysis (GSEA) using the list of DE genes between DCIS from Black and White women with a variety of gene sets but found no significantly enriched pathways (Supplementary Figure S2A showing Hallmark pathways without significant enrichment). Since we identified DE genes between DCIS from Black and White women, we hypothesized there could be significant differences in gene expression between cases and controls by SRR. To investigate, we performed differential gene expression analysis in cases with iBE within 5 years from treatment versus the rest as described previoulsy [19], but separately for Black and White women. The analysis of Black cases (*n* = 33) versus controls (*n* = 66) identified 266 differentially expressed genes (FDR < 0.05, Supplementary Table S2, Supplementary Figure S2B), whereas analysis of White cases (*n* = 51) versus controls (*n* = 140) identified 812 differentially expressed genes (FDR < 0.05, Supplementary Table S3, Supplementary Figure S2C).

**Fig. 2:**
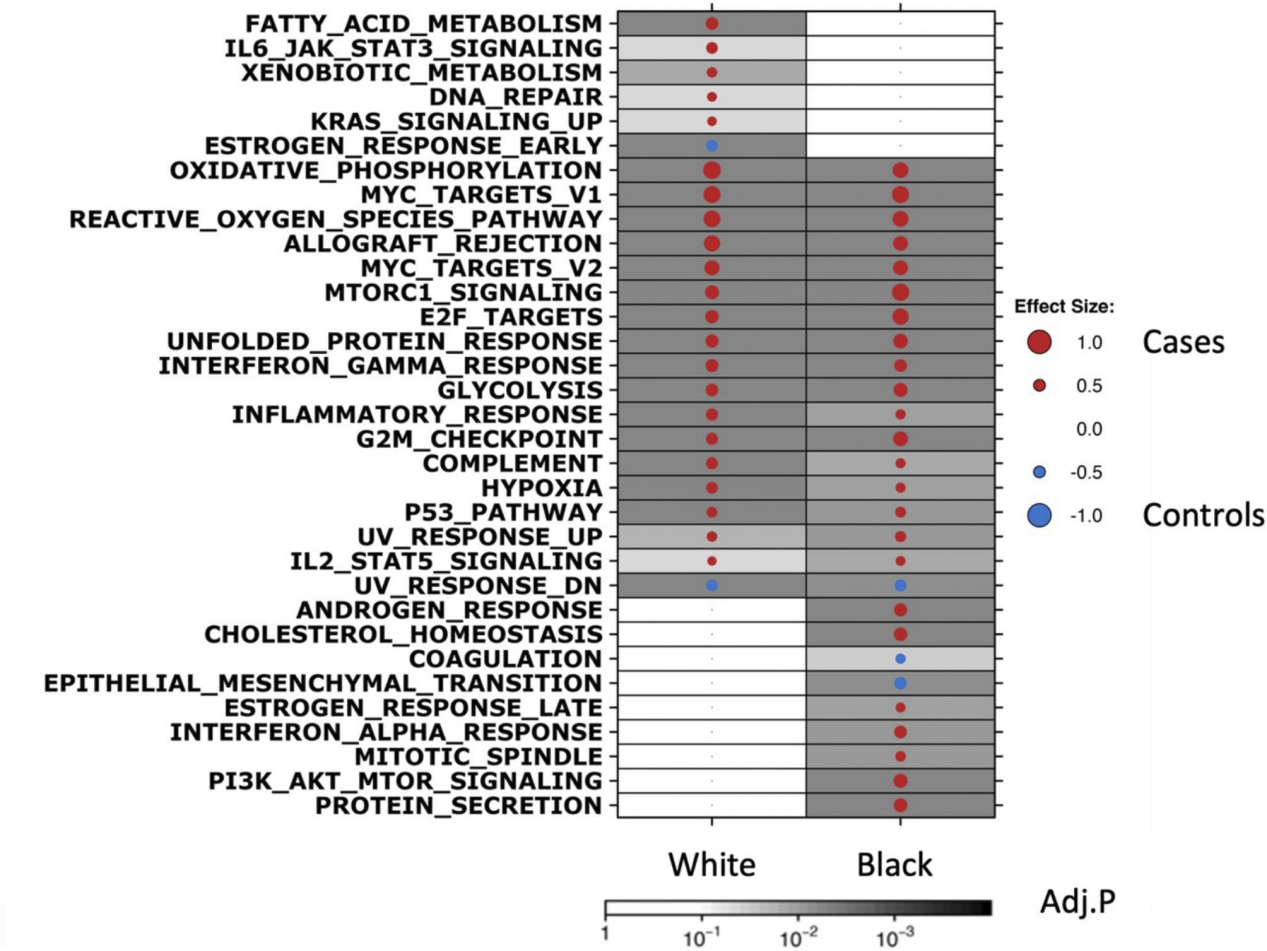
Gene Set Enrichment Analysis by SRR and outcome groups. GSEA Hallmark analysis of differentially expressed genes between DCIS from White cases vs. controls (left column) and Black cases vs. controls (right column), respectively. Dot size and color represent the magnitude and direction of pathway deregulation. Background shading indicates FDR. Effect size and FDR from GSEA algorithm

We performed GSEA using the Hallmark gene sets, which identified many common pathways associated with ipsilateral recurrence in both Black and White women, including Allograft Rejection, cell-cycle associated pathways (E2F Targets, G2M Checkpoint), pathways involved in metabolism (Oxidative Phosphorylation, Glycolysis), and mTORc1 signaling (Fig. 2). Interestingly however, we also observed pathways that were differentially enriched by SRR, such as increased expression of genes implicated in Fatty Acid Metabolism, IL6/JAK/STAT3 Signaling, and DNA Repair, which were associated with recurrence in White cases only. Conversely, increased Androgen Response and Interferon Alpha Signaling were among the pathways that were associated with recurrence in Black cases only. Finally, Estrogen Response pathways were significantly enriched in White controls and Black cases, further indicating fundamental pathways that may play have variable roles in biologic outcome, depending on SRR.

To further investigate differential pathway enrichment in iBEs by SRR, we performed GSEA with GO terms, KEGG, and REACTOME gene sets (Supplementary Figure S2D-F). We noted that pathways involved in immune response, such as Allograft Rejection (Hallmark, KEGG), Antigen Processing and Presentation (KEGG), and Natural Killer Cell Mediated Cytotoxicity (KEGG) were generally increased in cases, compared to controls, regardless of SRR. However, some immune-associated pathways were specifically enriched only in cases compared to controls in Black patients, such as Activation of Immune Response (GO), Immune Effector Processes (GO), Leukocyte Cell-Cell Adhesion (GO), and T-cell Receptor Signaling (KEGG). Conversely, Positive Regulation of T-cell Mediated Cytotoxicity (GO), Regulatory T-cell Differentiation (GO), and IL-6/JAK/STAT3 signaling (Hallmark) were increased only in cases compared to controls in White patients. These analyses further highlighted pathways that were differentially associated with 5-year outcome between DCIS from Black versus White women.

Together these results support the intriguing finding that different pathways distinguish cancer biology in Black and White patients along the spectrum of biologic processes, starting from the initiation of DCIS, and extending to recurrence and progression. In the race-stratified case-control analysis, 99 (37%) differentially expressed genes identified in the Black case-vs-control analysis, were represented in the previously identified HTAN DCIS classifier, whereas 357 (44%) of the 812 differentially expressed genes identified in the White case-vs-control analysis were included in the classifier. Correlation analysis of the effect size (ES) of the 812 genes included in the HTAN DCIS classifier in the Black case-control analysis and White case-control analysis showed significant correlation (*R* = 0.72, *P* < 2.2e-16, Supplementary Figure S2G), however the overall effect size from analysis in Black patients were attenuated compared to White patients. This analysis, combined with the worse outcome amongst Black women in the HTAN DCIS classifier Low-risk group (Fig. 1G) suggests genes associated with iBEs in Black women may be underrepresented in the classifier.

### Established biomarkers, cell type distribution, and genomic aberrations by SRR

**Fig. 3:**
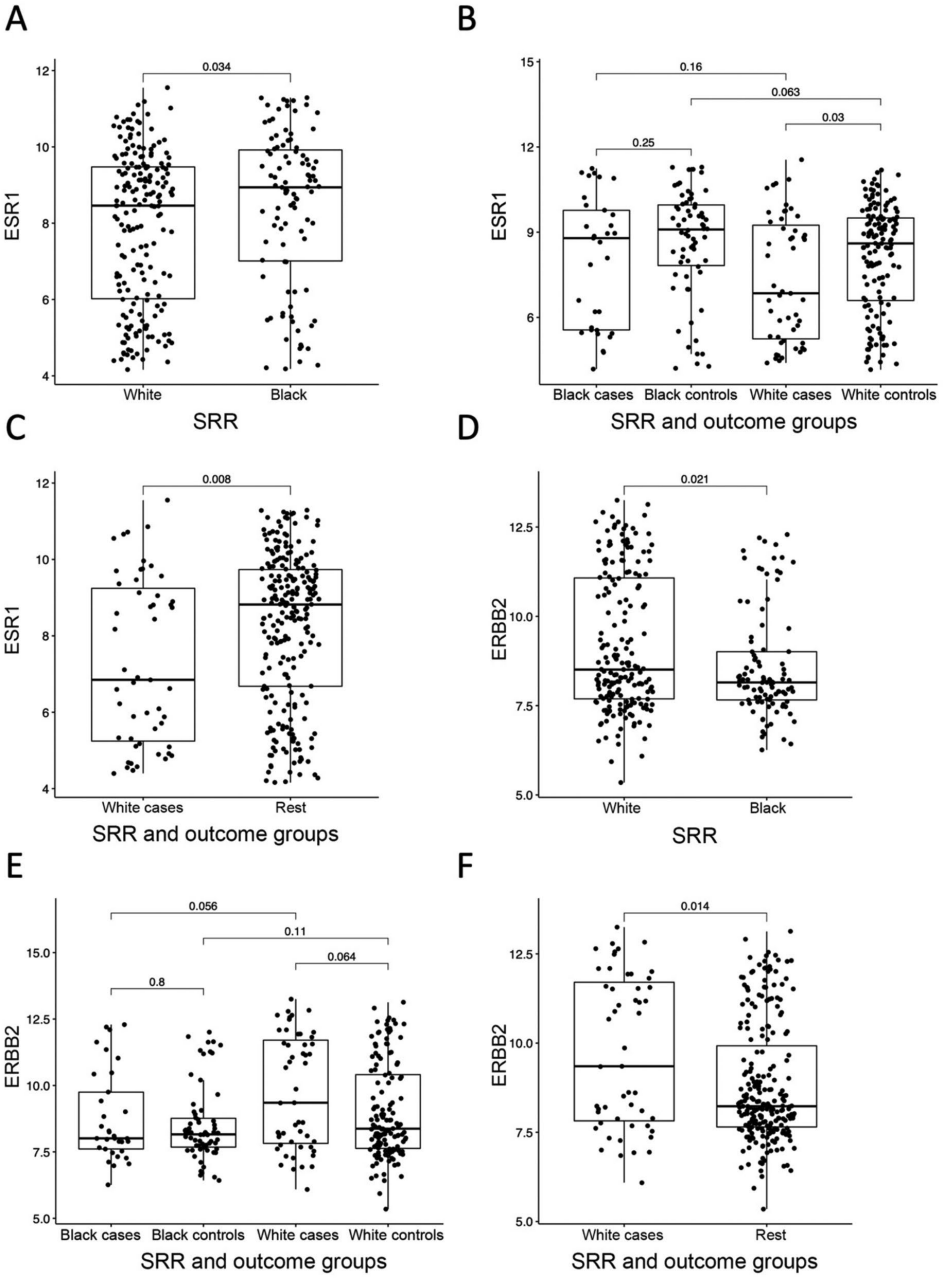
ER and HER2 expression by SRR and outcome groups. **A**) ER (*ESR1*) gene expression by SRR. **B**) ER (*ESR1*) gene expression by SRR and outcome groups. **C**) ER (*ESR1*) gene expression in White cases vs. Black cases and all controls combined. **D**) HER2 (*ERBB2*) gene expression by SRR. **E**) HER2 (*ERBB2*) gene expression by SRR and outcome groups. **F**) HER2 (*ERBB2*) gene expression in White cases vs. Black cases and all controls combined. **A-F**) Boxplots represent median, 0.25 and 0.75 quantiles with whiskers at 1.5x interquartile range. P-values from Wilcoxon rank-sum test

To further investigate molecular differences by race, we analyzed established IBC biomarkers by SRR, including gene expression of *ESR1* (ER) and *ERBB2* (HER2), as well as the intrinsic subtypes defined by PAM50 in invasive breast cancers. We first compared ESR1 expression in DCIS diagnosed in Black, compared to White women. ESR1 expression was significantly elevated in Black compared to White women (*P* = 0.034, Fig. 3A). Moreover, as indicated by GSEA (Fig. 2), we found a significant difference in ESR1 expression by SRR further stratified by outcome groups, with White cases having significantly lower ESR1 expression compared to White controls (*P* = 0.03, Fig. 3B). No significant difference in ESR1 expression was observed between Black cases and Black controls. Furthermore, White cases had significantly lower ESR1 expression compared to all other groups combined (Black cases and all controls *P* = 0.008, Fig. 3C). We then compared ERBB2 expression in DCIS diagnosed in Black, compared to White women. For ERBB2 expression we observed the opposite trend to that seen for ESR1, with significantly lower expression in Black, compared to White women (*P* = 0.021, Fig. 3D). Again, a difference by outcome was observed for White women only, with increased ERBB2 expression in White cases compared to white controls, although it did not reach statistical significance (*P* = 0.064, Fig. 3E). Moreover, White cases had significantly higher ERBB2 expression compared to all other groups combined (*P* = 0.014, Fig. 3F).

Next, we analyzed the association between PAM50 subtypes and SRR. We found no significant association between PAM50 and SRR only (*P* = 0.085, Supplementary Figure S3A), but identified a significant association for Black controls who were enriched for the Normal-like subtype (*P* = 0.016, Supplementary Figure S3B) in the analysis including SRR and outcome groups. We note that we observed no enrichment for Basal-like DCIS in Black women, despite these being reported as more common in IBC from Black/African women [11, 28–32]. This could indicate that the PAM50 Basal-like subtype may not be applicable to early-stage precancers such as DCIS [19, 33].

**Fig. 4:**
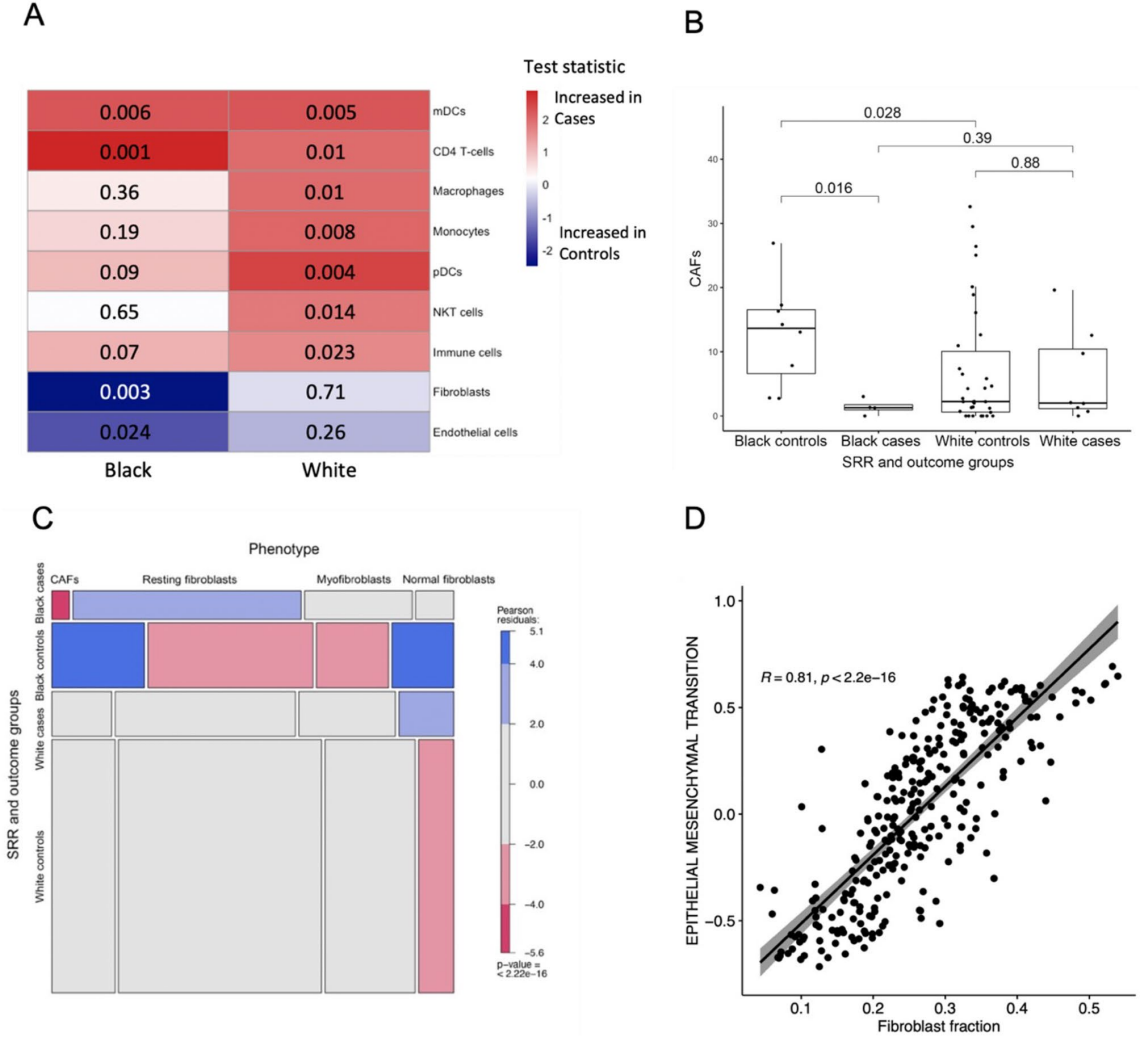
Cell type distribution by SRR and outcome groups. **A**) Heatmap of P-values (Wilcoxon rank-sum test) for inferred cell types in Black cases vs. controls (left column) and White cases vs. controls (right column). mDC = Myeloid dendritic cells. pDC = Plasmacytoid dendritic cells. **B**) Cancer associated fibroblasts (CAFs) distribution by SRR and outcome groups in MIBI sample-level data (*n* = 54). Boxplot represents median, 0.25 and 0.75 quantiles with whiskers at 1.5x interquartile range. P-values from Wilcoxon rank-sum test. **C**) Mosaic plot showing distribution of fibroblast phenotypes by SRR and outcome groups in MIBI single-cell data (*n* = 4926). P-value from Chi^2^ test. **D**) Correlation between EMT and fibroblast fraction by RNA-seq analysis in combined TBCRC and RAHBT cohorts, all samples regardless of SRR (*n* = 290). Correlation coefficient and P-value from Pearson correlation analysis

GSEA indicated an increased immune component in cases versus controls for both Black and White women (Fig. 2and Supplementary Figure S2D-F). To investigate differences in (immune) cell type composition, we leveraged available CibersortX data from the TBCRC and RAHBT cohorts [19]. We observed no significant difference in cell type distribution based on SRR (Supplementary Figure S4). However, cell type distribution by SRR and outcome groups revealed significant differences (Fig. 4A, Supplementary Figure S5A-O), with myeloid dendritic cells (mDCs) and CD4 T-cells significantly higher in both Black cases versus Black controls, and White cases versus White controls (ES_mDC, Black_=1.4, P_mDC, Black_=0.0056; ES_mDC, White_=1.18, P_mDC, White_=0.0052; ES_CD4, Black_=2.29, P_CD4, Black_=0.0013; ES_CD4, White=_1.63, P_CD4, White_=0.01).

Moreover, macrophages, monocytes, plasmacytoid dendritic cells (pDCs), NKT-cells, and overall immune cell population were significantly higher in White cases versus controls (ES_macro_=1.39, P_macro_=0.01; ES_mono_=2.39, P_mono_=0.0084; ES_pDC_=1.46, P_pDC_=0.0035; ES_NKT_=1.27, P_NKT_=0.0014, ES_immune_=1.24, P_immune_=0.023). For most of these, increased immune cell populations could be observed in Black cases versus controls, although they did not reach statistical significance. However, fibroblasts and endothelial cells showed significantly lower proportions in Black cases versus Black controls (ES_fibro_=0.83, P_fibro_=0.0032; ES_endo_=0.91, P_endo_=0.024), with samples from Black cases depleted of fibroblasts compared to all other SRR-outcome groups. Moreover, high levels of fibroblasts were associated with better 5-year outcome in Black (HR (95% CI): 0.004 (0.0001–0.16), *P* = 0.003), but not White women (HR (95% CI): 0.57 (0.032–10), *P* = 0.70).

To further investigate the differences in fibroblast population in Black cases versus other SRR-outcome groups, we used Multiplex Ion Beam Imaging (MIBI) protein expression data from the RAHBT cohort [19, 20]. Previous MIBI analysis identified four different fibroblast phenotypes, namely normal fibroblasts, myofibroblasts, resting fibroblasts, and cancer associated fibroblasts (CAFs) [20]. Using available sample-level MIBI data from 54 patients, we found that CAFs were significantly depleted in Black cases versus black controls (*P* = 0.016, Fig. 4B). None of the remaining fibroblast phenotypes or overall fibroblast population showed significant difference between SRR-stratified outcome groups (Supplementary Figure S5P-S). However, using single-cell level MIBI data, we found that the overall distribution of fibroblast subtypes differed between SRR-outcome groups, with Black cases depleted for CAFs but enriched for resting fibroblasts, and Black controls depleted for resting- and myofibroblasts while enriched for normal fibroblasts and CAFs. White cases were enriched, and White controls depleted, of normal fibroblasts (Fig. 4C). Finally, we performed single sample gene set variation analysis of the 34 Hallmark pathways in Fig. 2A. We correlated the enrichment score for each pathway in each sample to the respective sample’s fibroblast fraction. This analysis showed that the Epithelial Mesenchymal Transition (EMT) pathway and fibroblast fraction were highly correlated across all samples (*R* = 0.81, *P* < 2.2e-16, Fig. 4D). According to Hallmark GSEA (Fig. 2A), the EMT pathway was enriched in Black controls compared to cases, correlating with the observed enrichment of fibroblasts in these samples. These results indicate increased mesenchymal gene expression in DCIS samples from Black cases compared to Black controls. Taken together, our results further indicate that different cell abundances contribute to the observed outcome differences based on SRR.

Finally, we investigated differences by SRR on the genomic level by analyzing copy number variations (CNVs) by SRR and outcome groups (Table 2, Supplementary Figure S6). Amplification of 17q21.31 and 17q12, and deletion of 8p11.22, were significantly enriched in DCIS from White versus Black women (P_17q21.31_=0.0004, P_17q12_ = 0.025, P_8p11.22_=0.041, Table 2A, Supplementary Figure S6A-C).

**Table 2:** Significant CNVs by SRR (A) and SRR and outcome groups (B)

**A**				
CNVs	*P*=	Direction of prevalence		
Amp 17q21.31	**0.0004**	White women		
Amp 17q12	**0.0245**	White women		
Del 8p11.22	**0.0412**	White women		
**B**				
	White cases vs. controls	Direction of prevalence	Black cases vs. controls	Direction of prevalence
CNVs	**P=**		**P=**	
Del 1p21.3	**0.033**	controls	0.614	NA
Del 3p14.1	**0.009**	controls	0.913	NA

The amplification of 17q12 in DCIS from White women correlated with the observed increased ERBB2 expression in these lesions (Fig. 3D). Analysis of CNVs by SRR and outcome groups found that deletion of 1p21.3 (*P* = 0.033) and 3p14.1 (*P* = 0.009) were enriched in White controls compared to White cases (Table 2B, Supplementary Figure S6D, E). We found no significant differential CNVs between Black cases and controls. None of the CNVs identified here were significantly enriched in IBC by ancestry in TCGA data [32]. Finally, we investigated Percentage of the Genome Altered (PGA), overall amplifications and deletions by SRR only and SRR-outcome, with no significant difference between groups (Supplementary Figure S6F-K). Taken together, our results indicate that there are larger differences in DCIS by SRR on the transcriptomic than the genomic level.

## Discussion

We recently presented the HTAN DCIS Atlas which included molecular analyses of DCIS epithelium and microenvironment at the genomic, transcriptomic and proteomic levels for 774 DCIS samples from 542 patients. We identified epithelial and stromal subtypes specific to DCIS, and generated the HTAN DCIS risk classifier for recurrence. However this study did not take SRR or ancestry into account.

There has been an important and increasing focus on the need to investigate molecular differences between tumors based on ancestry, ethnicity, or race, with growing recognition of the implications of such findings for cancer screening, diagnosis, treatment, and follow up [34]. While race is a social construct, it is often associated with poor outcomes in part due to access to health care, social determinants of health, and racial inequity, and thus continues to offer incremental, useful information, including elucidation of health disparities [34–36]. It is well established that in clinical cohorts SRR and genetically inferred ancestry are concordant in that self-reported Black patients tend to exhibit higher inferred proportion of DNA of African ancestry. This was, as expected, recapitulated in an ordination analysis based on genetic data from a subset of patients from our study cohort with available low-pass whole-genome DNA sequencing. Given our objective of studying the effect of self-reported race, we opted to use self-reported race rather than genetically inferred ancestry, as this would require use of an arbitrary cutoff to categorize patients as either genetically African or European. Thus in our analysis, we have used the data that patients have self-reported, as is employed in routine clinical care settings.

Emerging evidence in breast and in other cancers suggests there are biologic disparities in outcome by race, underscoring the need to understand the differential pathways that could account for such differences. Historically, study cohorts have included few Black and Asian patients, or indeed have not collected information on ancestry, ethnicity, or race [37]. Thus, there is an unmet need to address disparities in IBC diagnosis and outcomes among Black women and other minorities, which can in part be addressed by better understanding key differences in tumor biology between White and Black women. Recent studies ae starting to address these knowledge gaps; in a recent analysis of the TAILORx trial of over 10,000 women with hormone receptor-positive, node-negative breast cancer, locoregional recurrence was significantly higher in Black women after adjustment for treatment, patient and tumor characteristics (HR = 1.78 (1.15–2.77)) compared to White women [38]confirming that there are latent features that are not measured by routine clinical variable that nevertheless impact important outcomes.

Here, we set out to investigate the molecular differences in DCIS based on SRR and ancestry by combining data from the two large DCIS cohorts from the HTAN DCIS Atlas. First, we generated global genetic ancestry calls from WGS data on a subset of the cohort, and compared these to SRR. As expected, this analysis showed exceptionally high concordance between SRR and genetic ancestry. By using SRR in the overall cohort analyses, we observed a significant outcome difference by race, with Black women having significantly shorter time to recurrence, and IBC recurrence specifically, compared to White women (Fig. 1B-C). While treatment modality was strongly associated with outcome, it could not fully explain the observed outcome disparity between Black and White women (Fig. 1D-E, Supplementary Figure S1C).

Next, we evaluated genomic differences between DCIS tumors from Black and White women, and while we observed few clear differentially expressed genes comparing racial groups, we observed significant differences relating to gene expression and pathway enrichment when stratifying the groups by clinical outcome (Fig. 2). Several pathways were associated with recurrence in Black but not White patients with DCIS, including Androgen Response, Interferon Alpha Response, and PI3K-AKT-MTOR Signaling. Conversely, DNA Repair, Fatty Acid- and Xenobiotic Metabolism, and KRAS Signaling were associated with recurrence in White women only. Notably, Estrogen Response Early and -Late was enriched in White controls compared to White cases, whereas Estrogen Response Late was enriched in Black cases versus Black controls (Fig. 2).

We further looked specifically at canonical breast cancer-related markers, and found that the ER- and HER2 + phenotypes were associated with recurrence events in White women only, contrary to what has been reported in IBC. The finding that ER expression was lower in DCIS from White compared to Black women (Fig. 3A) contrasts with results from analysis of IBC tumors from TCGA [32], but agrees with an analysis of SEER data where Black women had significantly more ER + DCIS compared to other racial/ethnic groups [11, 12]. With respect to ERBB2, we observed a significant enrichment of the 17q12 amplification in DCIS in White women, which correlated with the observed increased ERBB2 RNA expression in these samples (Fig. 3D). Notably, the 17q12 amplification was not differentially enriched between White cases and White controls, suggesting that the observed trend of increased ERBB2 expression in White cases (Fig. 3E) is caused by mechanisms other than amplification of the 17q12 locus, in line with previous reports on IBC [39]. These results are in contrast to results in invasive breast cancers from TCGA, which reported significantly more ER + tumors in women of European compared to women of African ancestry, with no difference in HER2 expression by ancestry [32].

We also evaluated PAM50, a multi-gene classifier which is highly prognostic in invasive cancers, and found no difference in the distribution of PAM50 subtypes in DCIS by SRR only. Of note, we found that Black controls were enriched for the Normal-like subtype. This is somewhat surprising, since Basal-like/triple negative (TNBC) IBC is more prevalent in Black women compared to White [11, 28–32]. Moreover, a study including more than 160,000 women reported that the risk of TNBC in Black women was almost twice that of White women after DCIS [11]. We and others have previously reported that the Basal-like subtype does not seem to apply fully to tumors at the DCIS stage [19, 33], which may in part explain this discrepancy, but also suggests that there are different pathways at play in the precancer, compared to invasive cancer settings.

Because DCIS is confined to the intraepithelial compartment, the DCIS microenvironment is very different from that of IBC and may reflect another important source of biologic discrimination in early breast neoplasia. In our cohort, inferred immune cells showed an overall trend of enrichment in cases versus controls regardless of SRR. In line with this, pathway analyses showed that several immune-related pathways were enriched in cases compared controls for both Black and White patients (Fig. 2A, Supplementary Figure S2D-F). Intriguingly, we found that low inferred fibroblast abundance was associated with recurrence within 5 years for Black women only (Fig. 4A, Supplementary Figure S4H). This contrasts with IBC studies, where fibroblasts, and CAFs in particular, are associated with greater propensity for aggression [40–44]. Recent studies suggested fibroblast abundance in DCIS contribute to invasive behavior [45, 46]. Importantly, none of these studies included race as a factor in the analyses. Also of note, by MIBI we here observed significantly reduced CAF levels in Black cases versus Black controls (Fig. 4B), albeit in a very small data set. Moreover, using RNA-seq data we found that fibroblast abundance was highly correlated with EMT enrichment (Fig. 4D).

According to American Cancer Society’s Breast Cancer Statistics 2022, Black women have slightly lower incidence of IBC than White women, but 40% higher IBC mortality rates [47]. This reflects in part that Black women have higher early incidence of TNBC and the poorest 5-year survival across race/ethnic groups within TNBC [47]. Several studies have searched for tumor biological features that can explain this discrepancy. One study found that IBC tumors from Black women had greater genetic heterogeneity and more basal gene expression, suggesting more aggressive tumor biology [31]. Another reported higher microvessel density and macrophage infiltration in IBC from Black women compared to White [48]. Huo et al. analyzed genomic, transcriptomic, proteomic and methylome data from IBCs in TCGA by ancestry. While they found many molecular differences between IBCs from women with African versus European ancestry, including CNVs, gene expression, and DNA methylation, most of the molecular differences were eliminated after adjusting for intrinsic subtype [32].

While there is compelling epidemiologic and clinical evidence about differences in the biology and behavior of IBC by race or ancestry, it is not clear to what extent the difference in outcome is due to biologic or socioeconomic factors related to racial inequity. Martini and colleagues investigated differential gene expression in women with TNBC based on both SRR and ancestry [49]. They found that genes uniquely associated with SRR were involved in pathways associated with lifestyle diseases, and in clustering analysis separated African American women from Ghanaians and Ethiopians. The authors hypothesized that these genes represent distinct environmental influences unique to African American patients, supporting the premise of social determinants linked to racial constructs.

The HTAN DCIS classifier was trained to predict iBE within 5 years from treatment. While we here showed that the classifier performs well in predicting 5-year iBEs regardless of SRR, we found significantly poorer outcome in the HTAN DCIS classifier low-risk group for Black compared to White women when including the full follow-up time (Fig. 1G). The observed difference was greatest > 10 years after initial treatment, which the classifier was not trained to detect. Liu et al. found that Black women had significantly higher risk of developing ipsilateral IBC away from the original DCIS lesion, as well as contralateral IBC, suggesting an underlying genetic susceptibility to IBC, early exposures, and/or interactions between these fundamental to breast tumors in Black women [11]. While our recurrence dataset included only ipsilateral events, one may speculate that the patients with recurrence > 10 years from treatment could be de-novo lesions not related to the original DCIS, as shown in clonality studies [50].

Despite the large and comprehensive dataset we used for our analysis, our study has several limitations. The combined cohort analyzed here consisted of 34% Black women, which is a large fraction compared to previous studies analyzing DCIS by race or ethnicity [6–12]. Nevertheless, our subgroup analyses were often based on relatively small sample sets, and future studies on larger, racially diverse cohorts may find additional differences. Moreover, our multivariable model does not account for account for potential interactions among the covariates, nor did it account for other potentially important observable or unobservable baseline risk factors. Other limiting factors are missing information on reproduction factors, lifestyle factors, and endocrine treatment, however we did note that in the TBCRC cohort, the use of ET was comparable between groups. In addition, the original cohorts included only 5 women identifying as Asian and a single Pacific islander, thus these groups were excluded from our analyses. Future studies investigating molecular differences in DCIS by SRR and ancestry should focus on including sizeable representations of other racial groups. Our cohort also did not include information on ethnicity, socioeconomic, or environmental factors, thus we were unable to evaluate their contribution to outcome disparity. Furthermore, race is often used as a surrogate for global genetic ancestry. Importantly, we noticed a broad range in genetic ancestry in both self-reported White and Black patients, which provides an important future opportunity to evaluate the role of local and global genetic ancestry in determining breast cancer biology.

## Conclusion

Our results suggest there are fundamental biologic differences related to cancer initiation, recurrence, and invasive progression in Black, compared to White women. Our study creates a foundation for future molecular and epidemiological studies to identify drivers that contribute to the racial differences in DCIS and IBC outcome, as well as opportunities to tailor prevention and treatment strategies according to relevant and racially variable pathways. Given the distinct nature of the disease and risk for recurrence based on SRR, evaluating risk predictors either based on established clinical elements such as receptor status or complex molecular parameters, such as the HTAN classifier, should seek to understand biology in the context of race and the racial composition of the population to be evaluated.

## Electronic supplementary material

Below is the link to the electronic supplementary material.

Supplementary Material 1

Supplementary Material 2

## Data Availability

We analyzed RNA and DNA sequencing, metadata, and MIBI data that are publicly available on the HTAN data portal (https://www.ncbi.nlm.nih.gov/projects/gap/cgi-bin/study.cgi?study_id=phs002371.v3.p1).

## Abbreviations

CAFs: Cancer-associated fibroblasts
CNVs: Copy-number variation
DCIS: Ductal carcinoma in situ
DE: Differentially expressed
EMT: Epithelial-mesenchymal transition
GSEA: Gene set enrichment analysis
HTAN: Human Tumor Atlas Network
IBC: Invasive breast cancer
iBE: Ipsilateral breast event
MIBI: Multiplex Ion Beam Imaging
PGA: Percentage of the Genome Altered
RAHBT: Resource of Archival Breast Tissue
SRR: Self-reported race
TBCRC: Translational Breast Cancer Research Consortium
TNBC: Triple-negative breast cancer
