# Correction: Analysis of ductal carcinoma in situ by self-reported race reveals molecular differences related to outcome

**DOI:** 10.1186/s13058-025-02106-6

**Published:** 2025-08-26

**Authors:** Siri H. Strand, Kathleen E. Houlahan, Vernal Branch, Lorraine M. King, Thomas Lynch, Belén Rivero-Guitiérrez, Bryan Harmon, Fergus Couch, Kristalyn Gallagher, Mark Kilgore, Shi Wei, Angela DeMichele, Tari King, Priscilla McAuliffe, Christina Curtis, Kouros Owzar, Jeffrey R. Marks, Graham A. Colditz, E. Shelley Hwang, Robert B. West

**Affiliations:** 1 Department of Pathology, Stanford University School of Medicine, Stanford, CA 94305 USA; 2 Stanford Cancer Institute, Stanford University School of Medicine, Stanford, CA 94305 USA; 3 Department of Medicine Genetics, Biomedical Data Science, Stanford University School of Medicine, Stanford, CA 94305 USA; 4 National Breast Cancer Coalition, 2001 L Street NW, Suite 500 PMB#50111, Washington, DC 20036 USA; 5 Department of Surgery, Duke University School of Medicine, Durham, NC 27708 USA; 6 Department of Pathology, Montefiore Medical Center, New York City, NY USA; 7 Department of Pathology, Mayo Clinic, Rochester, MN USA; 8 Department of Surgery, University of North Carolina at Chapel Hill, Chapel Hill, NC USA; 9 Department of Pathology, University of Washington, Seattle, WA USA; 10 Department of Pathology, University of Alabama at Birmingham, Birmingham, AL USA; 11 Department of Medicine, University of Pennsylvania, Philadelphia, PA USA; 12 Department of Surgery, Brigham and Women’s Hospital, Boston, MA USA; 13 Department of Surgery, University of Pittsburgh, Pittsburgh, PA USA; 14 Duke Cancer Institute, Duke University School of Medicine, Durham, NC 27708 USA; 15 Department of Biostatistics & Bioinformatics, Duke University School of Medicine, Durham, NC 27708 USA; 16 Department of Surgery, Washington University School of Medicine, St. Louis, MO 63110 USA

**Correction to: Strand et al. Breast Cancer Research (2024) 26:127**.

10.1186/s13058-024-01885-8.

Following the publication of the original article, the authors reported that Lorraine King’s name had been inadvertently omitted.

The name of Lorraine King has been added to the authorship list shown here.

Additionally, the Author’s contribution section has been corrected to reflect the change.

**Conceptualization:** ESH, RBW, GAC, JRM.

**Investigation:** SHS, KEH, and RBW.

**Resources:** LMK, BH, FC, KG, MK, SW, AD, TK, PM, BRG.

**Writing – Original Draft:** SHS and RBW.

**Writing – Review & Editing:** All co-authors.

**Funding Acquisition:** ESH, RBW, GAC.

**Supervision:** CC, GAC, ESH, and RB.

**Corresponding author:** Correspondence to Robert B. West.

The original article has been updated accordingly.

