# Supplementary material for: Analysis of ductal carcinoma in situ by self-reported race reveals molecular differences related to outcome
Source: Breast Cancer Res. 2024 Sep 2;26:127. doi: 10.1186/s13058-024-01885-8 (PMC11367816; doi:10.1186/s13058-024-01885-8)
Supplement: Supplementary file 2 — Supplementary Material 2 [file 13058_2024_1885_MOESM2_ESM.pdf]

Figure S1

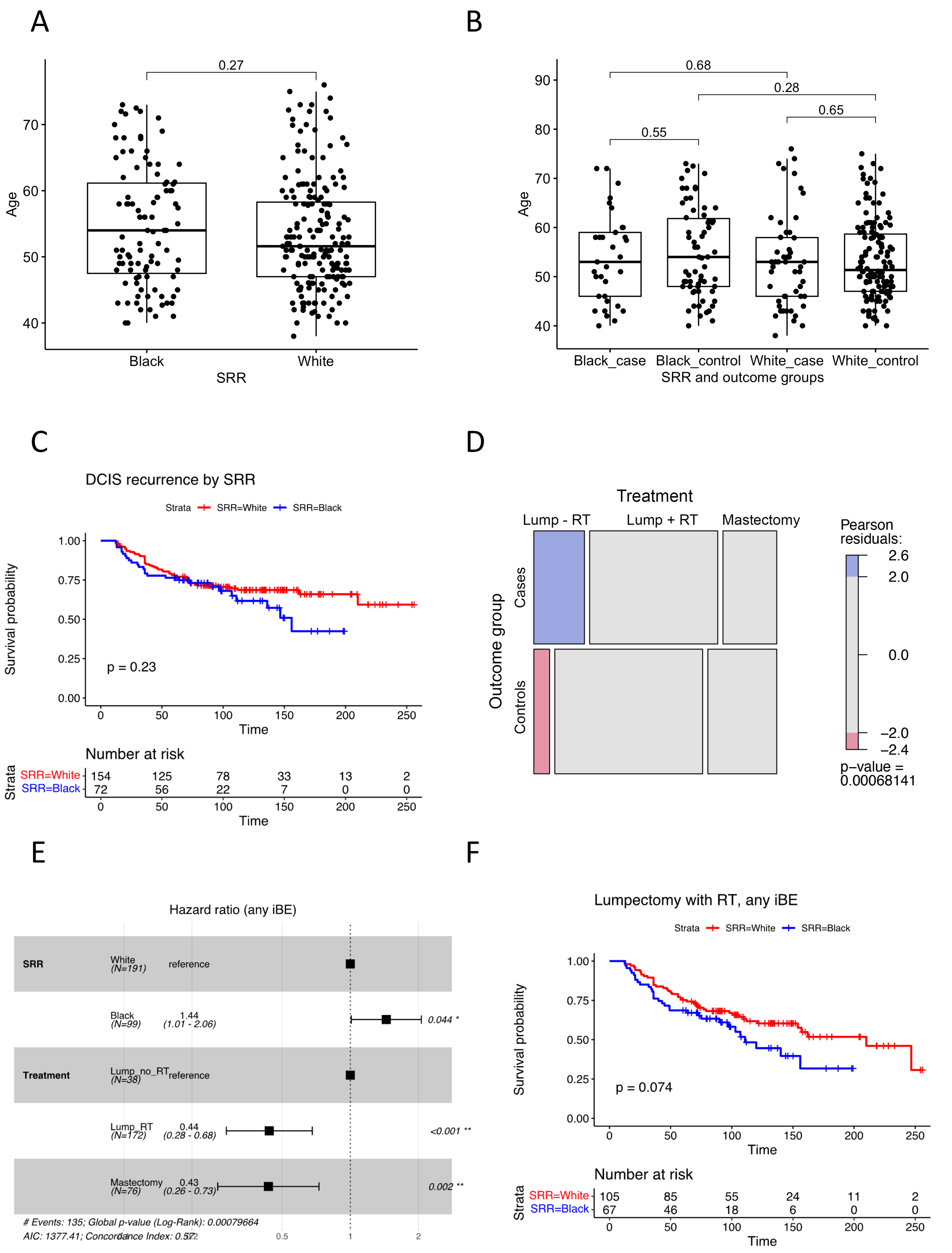
A
0.27
Age
70
60
50
40
Black
White
SRR
B
0.68
0.28
0.65
0.55
90
80
Black_case
Black_control
White_case
White_control
SRR and outcome groups
C
DCIS recurrence by SRR
Strata
SRR=White
SRR=Black
Survival probability
1.00
0.75
0.50
0.25
0.00
p = 0.23
0
50
100
150
200
250
Time
Number at risk
SRR=White 154 125 78 33 13 2
SRR=Black 72 56 22 7 0 0
D
Treatment
Lump - RT
Lump + RT
Mastectomy
Outcome group
Cases
Controls
Pearson residuals:
2.6
2.0
0.0
−2.0
−2.4
p−value = 0.00068141
E
Hazard ratio (any iBE)
SRR
White (N=191) reference
Black (N=99) 1.44 (1.01 - 2.06) 0.044 *
Treatment
Lump_no_RT (N=38) reference
Lump_RT (N=172) 0.44 (0.28 - 0.68) <0.001 **
Mastectomy (N=76) 0.43 (0.26 - 0.73) 0.002 **
Events: 135; Global p-value (Log-Rank): 0.00079664
AIC: 1377.41; Concordance Index: 0.57
0.5
1
2
F
Lumpectomy with RT, any iBE
p = 0.074
SRR=White 105 85 55 24 11 2
SRR=Black 67 46 18 6 0 0

Figure S1

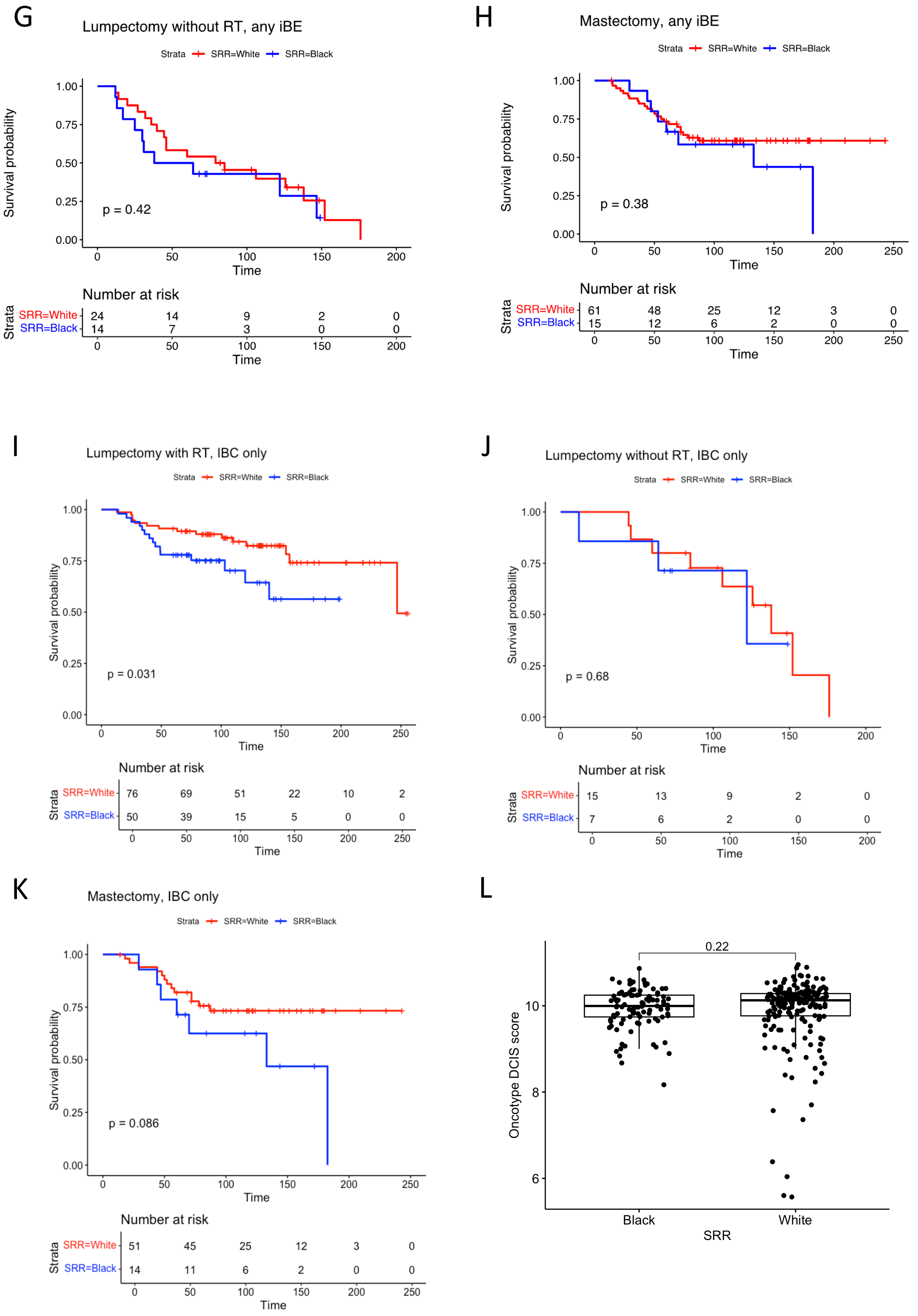
G
Lumpectomy without RT, any iBE
Strata SRR=White SRR=Black
Survival probability
1.00
0.75
0.50
0.25
0.00
p = 0.42
0 50 100 150 200
Time
Number at risk
Strata
SRR=White 24 14 9 2 0
SRR=Black 14 7 3 0 0
0 50 100 150 200
Time
H
Mastectomy, any iBE
Strata SRR=White SRR=Black
Survival probability
1.00
0.75
0.50
0.25
0.00
p = 0.38
0 50 100 150 200 250
Time
Number at risk
Strata
SRR=White 61 48 25 12 3 0
SRR=Black 15 12 6 2 0 0
0 50 100 150 200 250
Time
I
Lumpectomy with RT, IBC only
Strata SRR=White SRR=Black
Survival probability
1.00
0.75
0.50
0.25
0.00
p = 0.031
0 50 100 150 200 250
Time
Number at risk
Strata
SRR=White 76 69 51 22 10 2
SRR=Black 50 39 15 5 0 0
0 50 100 150 200 250
Time
J
Lumpectomy without RT, IBC only
Strata SRR=White SRR=Black
Survival probability
1.00
0.75
0.50
0.25
0.00
p = 0.68
0 50 100 150 200
Time
Number at risk
Strata
SRR=White 15 13 9 2 0
SRR=Black 7 6 2 0 0
0 50 100 150 200
Time
K
Mastectomy, IBC only
Strata SRR=White SRR=Black
Survival probability
1.00
0.75
0.50
0.25
0.00
p = 0.086
0 50 100 150 200 250
Time
Number at risk
Strata
SRR=White 51 45 25 12 3 0
SRR=Black 14 11 6 2 0 0
0 50 100 150 200 250
Time
L
0.22
Oncotype DCIS score
10
8
6
Black
White
SRR

M

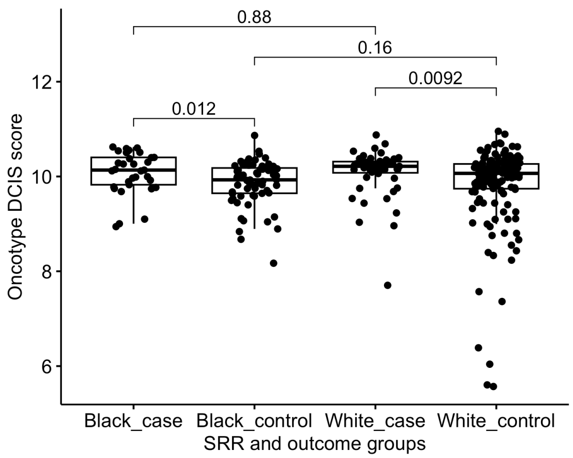

0.88
0.16
0.0092
0.012
Oncotype DCIS score
12
10
8
6
Black_case
Black_control
White_case
White_control
SRR and outcome groups

N

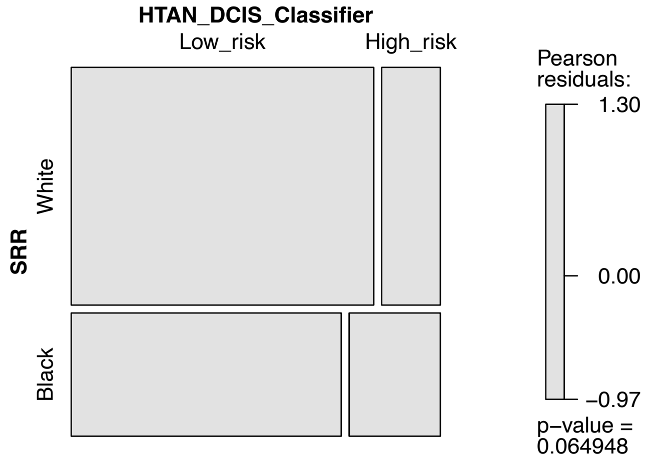

HTAN_DCIS_Classifier
Low_risk
High_risk
SRR
White
Black
Pearson residuals:
1.30
0.00
−0.97
p−value = 0.064948

O

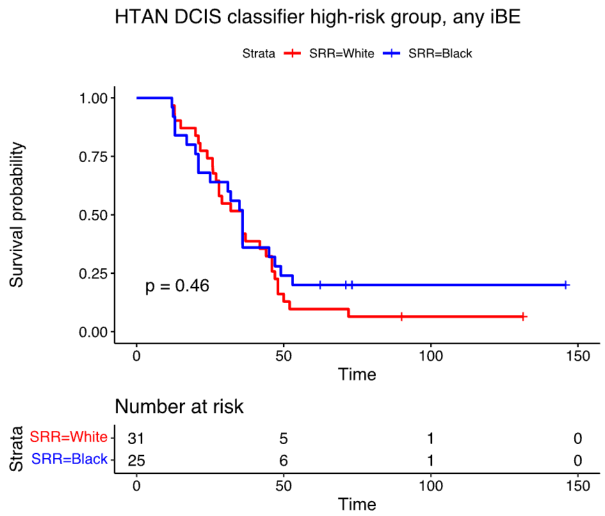

HTAN DCIS classifier high-risk group, any iBE
Strata
SRR=White
SRR=Black
Survival probability
1.00
0.75
0.50
0.25
0.00
p = 0.46
0
50
100
150
Time
Number at risk
Strata
SRR=White
SRR=Black
31
5
1
0
25
6
1
0

Figure S1: Outcome analysis by SRR

A) Distribution of age at diagnosis by SRR. B) Distribution of age at diagnosis by SRR and outcome groups. C) Kaplan-Meier plot of time to DCIS recurrence only (full follow-up) stratified by SRR. D) Mosaic plot showing distribution of treatment type by outcome groups. P-value from Chi^2 test. E) Forest plot of multivariable Cox regression analysis including SRR and treatment type, for any iBE (full follow-up). F) Kaplan-Meier plot of time to any iBE (full follow-up) for patients treated by lumpectomy and radiation treatment stratified by SRR. G) Kaplan-Meier plot of time to any iBE (full follow-up) for patients treated by lumpectomy without radiation treatment stratified by SRR. H) Kaplan-Meier plot of time to any iBE (full follow-up) for patients treated by mastectomy stratified by SRR. I) Kaplan-Meier plot of time to IBC only (full follow-up) for patients treated by lumpectomy and radiation treatment stratified by SRR. J) Kaplan-Meier plot of time to IBC only (full follow-up) for patients treated by lumpectomy without radiation treatment stratified by SRR. K) Kaplan-Meier plot of time to IBC only (full follow-up) for patients treated by mastectomy stratified by SRR. L) Distribution of Oncotype DX DCIS score calculated from RNA-seq data by SRR. M) Distribution of Oncotype DX DCIS score calculated from RNA-seq data by SRR and outcome data. N) Mosaic plot showing distribution of HTAN DCIS classifier risk by SRR. P-value from Chi^2 test. O) Kaplan-Meier plot of time to iBE (full follow-up) in the HTAN DCIS classifier high-risk group stratified by SRR. A, D, E, F, H, I, J, K) P-values from log-rank tests. A, B, L, M) Boxplots represent median, 0.25 and 0.75 quantiles with whiskers at 1.5x interquartile range. P-values from Wilcoxon rank-sum test.

Figure S2

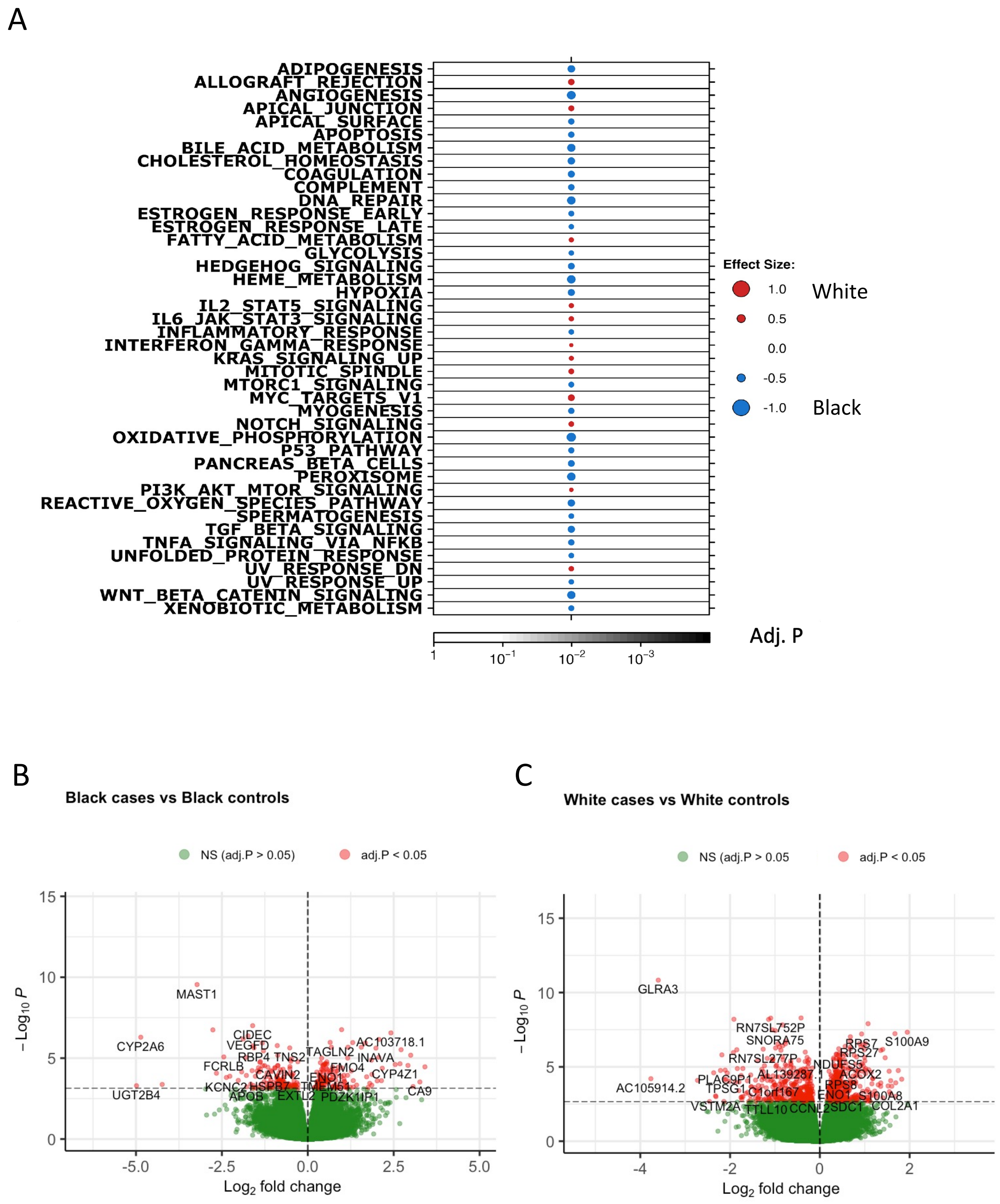
A
ADIPOGENESIS
ALLOGRAFT_REJECTION
ANGIOGENESIS
APICAL_JUNCTION
APICAL_SURFACE
APOPTOSIS
BILE_ACID_METABOLISM
CHOLESTEROL_HOMEOSTASIS
COAGULATION
COMPLEMENT
DNA_REPAIR
ESTROGEN_RESPONSE_EARLY
ESTROGEN_RESPONSE_LATE
FATTY_ACID_METABOLISM
GLYCOLYSIS
HEDGEHOG_SIGNALING
HEME_METABOLISM
HYPOXIA
IL2_STAT5_SIGNALING
IL6_JAK_STAT3_SIGNALING
INFLAMMATORY_RESPONSE
INTERFERON_GAMMA_RESPONSE
KRAS_SIGNALING_UP
MITOTIC_SPINDLE
MTORC1_SIGNALING
MYC_TARGETS_V1
MYOGENESIS
NOTCH_SIGNALING
OXIDATIVE_PHOSPHORYLATION
P53_PATHWAY
PANCREAS_BETA_CELLS
PEROXISOME
PI3K_AKT_MTOR_SIGNALING
REACTIVE_OXYGEN_SPECIES_PATHWAY
SPERMATOGENESIS
TGF_BETA_SIGNALING
TNFA_SIGNALING_VIA_NFKB
UNFOLDED_PROTEIN_RESPONSE
UV_RESPONSE_DN
UV_RESPONSE_UP
WNT_BETA_CATENIN_SIGNALING
XENOBIOTIC_METABOLISM
Effect Size:
1.0 White
0.5
0.0
-0.5
-1.0 Black
Adj. P
1
10^{-1}
10^{-2}
10^{-3}
B
Black cases vs Black controls
NS (adj.P > 0.05)
adj.P < 0.05
$-\log_{10} P$
$\log_2$ fold change
MAST1
CYP2A6
UGT2B4
CIDEC
VEGFD
RBP4
TNS2
FCRLB
CAVIN2
KCNC2
HSPB7
APOB
EXTL2
TAGLN2
ENO1
TMEM51
FMO4
AC103718.1
INAVA
CYP4Z1
CA9
PDZK1IP1
C
White cases vs White controls
NS (adj.P > 0.05
adj.P < 0.05
$-\log_{10} P$
$\log_2$ fold change
GLRA3
AC105914.2
RN7SL752P
SNORA75
RN7SL277P
AL139287.1
PLAC9P1
TPSG1
C1orf167
VSTM2A
TTLL10
CCNL2
SDC1
NDUFS5
RPS7
S100A9
RPS27
ACOX2
RPS8
ENO1
S100A8
COL2A1

Figure S2

D

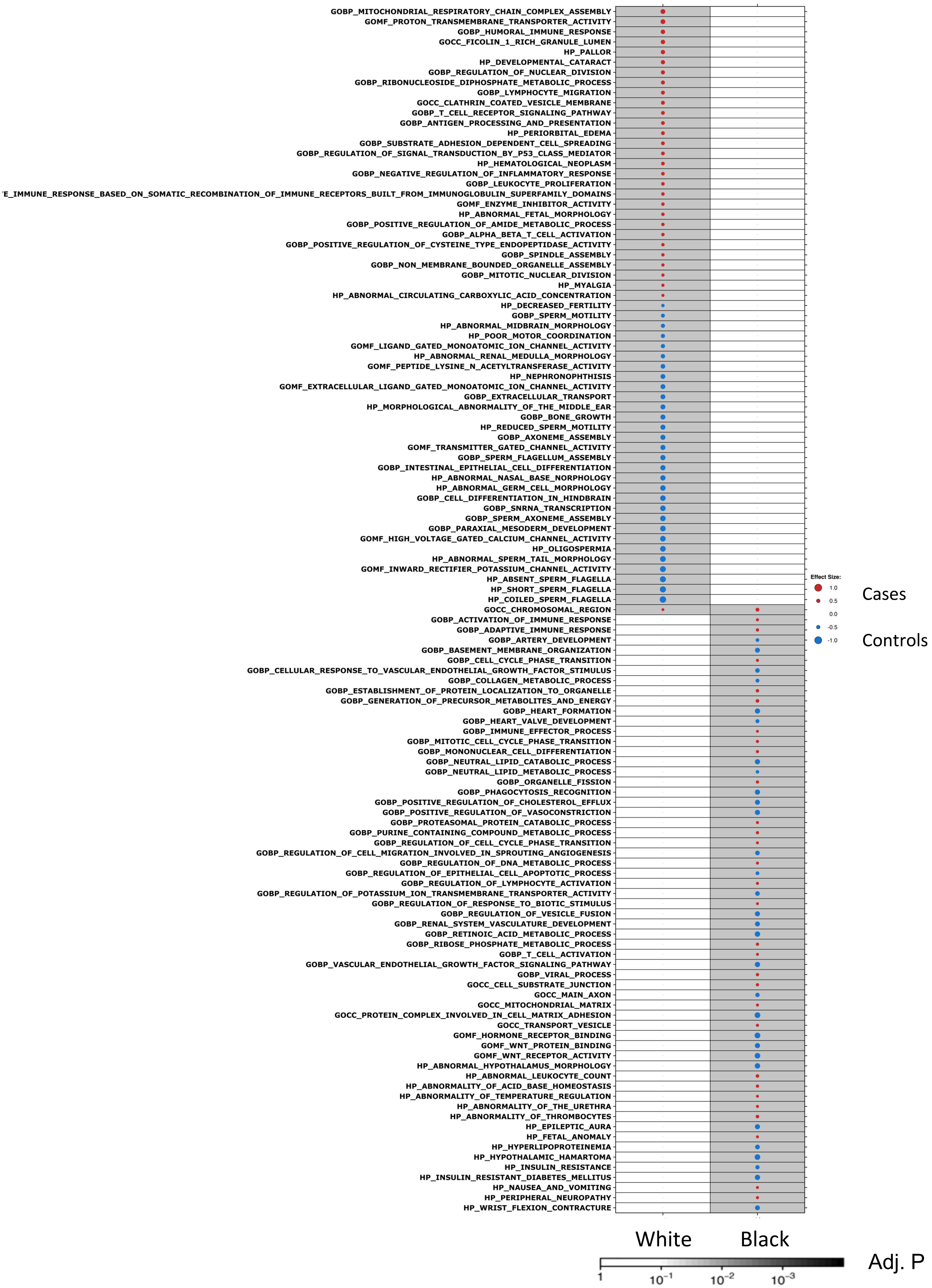

GOBP_MITOCHONDRIAL_RESPIRATORY_CHAIN_COMPLEX_ASSEMBLY
GOMF_PROTON_TRANSMEMBRANE_TRANSPORTER_ACTIVITY
GOBP_HUMORAL_IMMUNE_RESPONSE
GOCC_FICOLIN_1_RICH_GRANULE_LUMEN
HP_PALLOR
HP_DEVELOPMENTAL_CATARACT
GOBP_REGULATION_OF_NUCLEAR_DIVISION
GOBP_RIBONUCLEOSIDE_DIPHOSPHATE_METABOLIC_PROCESS
GOBP_LYMPHOCYTE_MIGRATION
GOCC_CLATHRIN_COATED_VESICLE_MEMBRANE
GOBP_T_CELL_RECEPTOR_SIGNALING_PATHWAY
GOBP_ANTIGEN_PROCESSING_AND_PRESENTATION
HP_PERIORBITAL_EDEMA
GOBP_SUBSTRATE_ADHESION_DEPENDENT_CELL_SPREADING
GOBP_REGULATION_OF_SIGNAL_TRANSDUCTION_BY_P53_CLASS_MEDIATOR
HP_HEMATOLOGICAL_NEOPLASM
GOBP_NEGATIVE_REGULATION_OF_INFLAMMATORY_RESPONSE
GOBP_LEUKOCYTE_PROLIFERATION
E_IMMUNE_RESPONSE_BASED_ON_SOMATIC_RECOMBINATION_OF_IMMUNE_RECEPTORS_BUILT_FROM_IMMUNOGLOBULIN_SUPERFAMILY_DOMAINS
GOMF_ENZYME_INHIBITOR_ACTIVITY
HP_ABNORMAL_FETAL_MORPHOLOGY
GOBP_POSITIVE_REGULATION_OF_AMIDE_METABOLIC_PROCESS
GOBP_ALPHA_BETA_T_CELL_ACTIVATION
GOBP_POSITIVE_REGULATION_OF_CYSTEINE_TYPE_ENDOPEPTIDASE_ACTIVITY
GOBP_SPINDLE_ASSEMBLY
GOBP_NON_MEMBRANE_BOUNDED_ORGANELLE_ASSEMBLY
GOBP_MITOTIC_NUCLEAR_DIVISION
HP_MYALGIA
HP_ABNORMAL_CIRCULATING_CARBOXYLIC_ACID_CONCENTRATION
HP_DECREASED_FERTILITY
GOBP_SPERM_MOTILITY
HP_ABNORMAL_MIDBRAIN_MORPHOLOGY
HP_POOR_MOTOR_COORDINATION
GOMF_LIGAND_GATED_MONOATOMIC_ION_CHANNEL_ACTIVITY
HP_ABNORMAL_RENAL_MEDULLA_MORPHOLOGY
GOMF_PEPTIDE_LYSINE_N_ACETYLTRANSFERASE_ACTIVITY
HP_NEPHRONOPHTHISIS
GOMF_EXTRACELLULAR_LIGAND_GATED_MONOATOMIC_ION_CHANNEL_ACTIVITY
GOBP_EXTRACELLULAR_TRANSPORT
HP_MORPHOLOGICAL_ABNORMALITY_OF_THE_MIDDLE_EAR
GOBP_BONE_GROWTH
HP_REDUCED_SPERM_MOTILITY
GOBP_AXONEME_ASSEMBLY
GOMF_TRANSMITTER_GATED_CHANNEL_ACTIVITY
GOBP_SPERM_FLAGELLUM_ASSEMBLY
GOBP_INTESTINAL_EPITHELIAL_CELL_DIFFERENTIATION
HP_ABNORMAL_NASAL_BASE_NORPHOLOGY
HP_ABNORMAL_GERM_CELL_MORPHOLOGY
GOBP_CELL_DIFFERENTIATION_IN_HINDBRAIN
GOBP_SNRNA_TRANSCRIPTION
GOBP_SPERM_AXONEME_ASSEMBLY
GOBP_PARAXIAL_MESODERM_DEVELOPMENT
GOMF_HIGH_VOLTAGE_GATED_CALCIUM_CHANNEL_ACTIVITY
HP_OLIGOSPERMIA
HP_ABNORMAL_SPERM_TAIL_MORPHOLOGY
GOMF_INWARD_RECTIFIER_POTASSIUM_CHANNEL_ACTIVITY
HP_ABSENT_SPERM_FLAGELLA
HP_SHORT_SPERM_FLAGELLA
HP_COILED_SPERM_FLAGELLA
GOCC_CHROMOSOMAL_REGION
GOBP_ACTIVATION_OF_IMMUNE_RESPONSE
GOBP_ADAPTIVE_IMMUNE_RESPONSE
GOBP_ARTERY_DEVELOPMENT
GOBP_BASEMENT_MEMBRANE_ORGANIZATION
GOBP_CELL_CYCLE_PHASE_TRANSITION
GOBP_CELLULAR_RESPONSE_TO_VASCULAR_ENDOTHELIAL_GROWTH_FACTOR_STIMULUS
GOBP_COLLAGEN_METABOLIC_PROCESS
GOBP_ESTABLISHMENT_OF_PROTEIN_LOCALIZATION_TO_ORGANELLE
GOBP_GENERATION_OF_PRECURSOR_METABOLITES_AND_ENERGY
GOBP_HEART_FORMATION
GOBP_HEART_VALVE_DEVELOPMENT
GOBP_IMMUNE_EFFECTOR_PROCESS
GOBP_MITOTIC_CELL_CYCLE_PHASE_TRANSITION
GOBP_MONONUCLEAR_CELL_DIFFERENTIATION
GOBP_NEUTRAL_LIPID_CATABOLIC_PROCESS
GOBP_NEUTRAL_LIPID_METABOLIC_PROCESS
GOBP_ORGANELLE_FISSION
GOBP_PHAGOCYTOSIS_RECOGNITION
GOBP_POSITIVE_REGULATION_OF_CHOLESTEROL_EFFLUX
GOBP_POSITIVE_REGULATION_OF_VASOCONSTRICTION
GOBP_PROTEASOMAL_PROTEIN_CATABOLIC_PROCESS
GOBP_PURINE_CONTAINING_COMPOUND_METABOLIC_PROCESS
GOBP_REGULATION_OF_CELL_CYCLE_PHASE_TRANSITION
GOBP_REGULATION_OF_CELL_MIGRATION_INVOLVED_IN_SPROUTING_ANGIOGENESIS
GOBP_REGULATION_OF_DNA_METABOLIC_PROCESS
GOBP_REGULATION_OF_EPITHELIAL_CELL_APOPTOTIC_PROCESS
GOBP_REGULATION_OF_LYMPHOCYTE_ACTIVATION
GOBP_REGULATION_OF_POTASSIUM_ION_TRANSMEMBRANE_TRANSPORTER_ACTIVITY
GOBP_REGULATION_OF_RESPONSE_TO_BIOTIC_STIMULUS
GOBP_REGULATION_OF_VESICLE_FUSION
GOBP_RENAL_SYSTEM_VASCULATURE_DEVELOPMENT
GOBP_RETINOIC_ACID_METABOLIC_PROCESS
GOBP_RIBOSE_PHOSPHATE_METABOLIC_PROCESS
GOBP_T_CELL_ACTIVATION
GOBP_VASCULAR_ENDOTHELIAL_GROWTH_FACTOR_SIGNALING_PATHWAY
GOBP_VIRAL_PROCESS
GOCC_CELL_SUBSTRATE_JUNCTION
GOCC_MAIN_AXON
GOCC_MITOCHONDRIAL_MATRIX
GOCC_PROTEIN_COMPLEX_INVOLVED_IN_CELL_MATRIX_ADHESION
GOCC_TRANSPORT_VESICLE
GOMF_HORMONE_RECEPTOR_BINDING
GOMF_WNT_PROTEIN_BINDING
GOMF_WNT_RECEPTOR_ACTIVITY
HP_ABNORMAL_HYPOTHALAMUS_MORPHOLOGY
HP_ABNORMAL_LEUKOCYTE_COUNT
HP_ABNORMALITY_OF_ACID_BASE_HOMEOSTASIS
HP_ABNORMALITY_OF_TEMPERATURE_REGULATION
HP_ABNORMALITY_OF_THE_URETHRA
HP_ABNORMALITY_OF_THROMBOCYTES
HP_EPILEPTIC_AURA
HP_FETAL_ANOMALY
HP_HYPERLIPOPROTEINEMIA
HP_HYPOTHALAMIC_HAMARTOMA
HP_INSULIN_RESISTANCE
HP_INSULIN_RESISTANT_DIABETES_MELLITUS
HP_NAUSEA_AND_VOMITING
HP_PERIPHERAL_NEUROPATHY
HP_WRIST_FLEXION_CONTRACTURE
Effect Size:
1.0
0.5
0.0
-0.5
-1.0
Cases
Controls
White
Black
Adj. P
1
10^-1
10^-2
10^-3

Figure S2

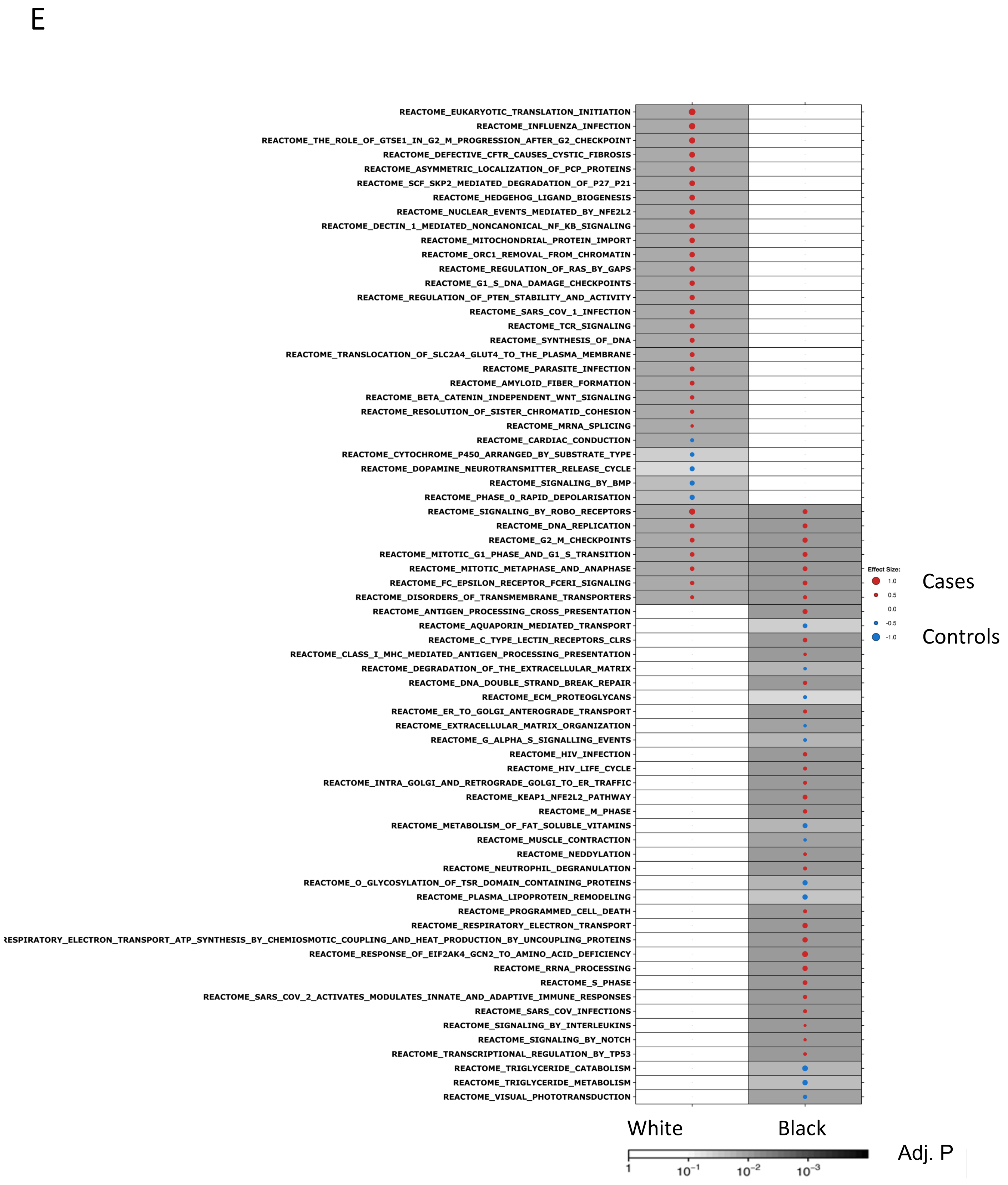

E
REACTOME_EUKARYOTIC_TRANSLATION_INITIATION
REACTOME_INFLUENZA_INFECTION
REACTOME_THE_ROLE_OF_GTSE1_IN_G2_M_PROGRESSION_AFTER_G2_CHECKPOINT
REACTOME_DEFECTIVE_CFTR_CAUSES_CYSTIC_FIBROSIS
REACTOME_ASYMMETRIC_LOCALIZATION_OF_PCP_PROTEINS
REACTOME_SCF_SKP2_MEDIATED_DEGRADATION_OF_P27_P21
REACTOME_HEDGEHOG_LIGAND_BIOGENESIS
REACTOME_NUCLEAR_EVENTS_MEDIATED_BY_NFE2L2
REACTOME_DECTIN_1_MEDIATED_NONCANONICAL_NF_KB_SIGNALING
REACTOME_MITOCHONDRIAL_PROTEIN_IMPORT
REACTOME_ORC1_REMOVAL_FROM_CHROMATIN
REACTOME_REGULATION_OF_RAS_BY_GAPS
REACTOME_G1_S_DNA_DAMAGE_CHECKPOINTS
REACTOME_REGULATION_OF_PTEN_STABILITY_AND_ACTIVITY
REACTOME_SARS_COV_1_INFECTION
REACTOME_TCR_SIGNALING
REACTOME_SYNTHESIS_OF_DNA
REACTOME_TRANSLOCATION_OF_SLC2A4_GLUT4_TO_THE_PLASMA_MEMBRANE
REACTOME_PARASITE_INFECTION
REACTOME_AMYLOID_FIBER_FORMATION
REACTOME_BETA_CATENIN_INDEPENDENT_WNT_SIGNALING
REACTOME_RESOLUTION_OF_SISTER_CHROMATID_COHESION
REACTOME_MRNA_SPLICING
REACTOME_CARDIAC_CONDUCTION
REACTOME_CYTOCHROME_P450_ARRANGED_BY_SUBSTRATE_TYPE
REACTOME_DOPAMINE_NEUROTRANSMITTER_RELEASE_CYCLE
REACTOME_SIGNALING_BY_BMP
REACTOME_PHASE_0_RAPID_DEPOLARISATION
REACTOME_SIGNALING_BY_ROBO_RECEPTORS
REACTOME_DNA_REPLICATION
REACTOME_G2_M_CHECKPOINTS
REACTOME_MITOTIC_G1_PHASE_AND_G1_S_TRANSITION
REACTOME_MITOTIC_METAPHASE_AND_ANAPHASE
REACTOME_FC_EPSILON_RECEPTOR_FCERI_SIGNALING
REACTOME_DISORDERS_OF_TRANSMEMBRANE_TRANSPORTERS
REACTOME_ANTIGEN_PROCESSING_CROSS_PRESENTATION
REACTOME_AQUAPORIN_MEDIATED_TRANSPORT
REACTOME_C_TYPE_LECTIN_RECEPTORS_CLRS
REACTOME_CLASS_I_MHC_MEDIATED_ANTIGEN_PROCESSING_PRESENTATION
REACTOME_DEGRADATION_OF_THE_EXTRACELLULAR_MATRIX
REACTOME_DNA_DOUBLE_STRAND_BREAK_REPAIR
REACTOME_ECM_PROTEOGLYCANS
REACTOME_ER_TO_GOLGI_ANTEROGRADE_TRANSPORT
REACTOME_EXTRACELLULAR_MATRIX_ORGANIZATION
REACTOME_G_ALPHA_S_SIGNALLING_EVENTS
REACTOME_HIV_INFECTION
REACTOME_HIV_LIFE_CYCLE
REACTOME_INTRA_GOLGI_AND_RETROGRADE_GOLGI_TO_ER_TRAFFIC
REACTOME_KEAP1_NFE2L2_PATHWAY
REACTOME_M_PHASE
REACTOME_METABOLISM_OF_FAT_SOLUBLE_VITAMINS
REACTOME_MUSCLE_CONTRACTION
REACTOME_NEDDYLATION
REACTOME_NEUTROPHIL_DEGRANULATION
REACTOME_O_GLYCOSYLATION_OF_TSR_DOMAIN_CONTAINING_PROTEINS
REACTOME_PLASMA_LIPOPROTEIN_REMODELING
REACTOME_PROGRAMMED_CELL_DEATH
REACTOME_RESPIRATORY_ELECTRON_TRANSPORT
RESPIRATORY_ELECTRON_TRANSPORT_ATP_SYNTHESIS_BY_CHEMIOSMOTIC_COUPLING_AND_HEAT_PRODUCTION_BY_UNCOUPLING_PROTEINS
REACTOME_RESPONSE_OF_EIF2AK4_GCN2_TO_AMINO_ACID_DEFICIENCY
REACTOME_RRNA_PROCESSING
REACTOME_S_PHASE
REACTOME_SARS_COV_2_ACTIVATES_MODULATES_INNATE_AND_ADAPTIVE_IMMUNE_RESPONSES
REACTOME_SARS_COV_INFECTIONS
REACTOME_SIGNALING_BY_INTERLEUKINS
REACTOME_SIGNALING_BY_NOTCH
REACTOME_TRANSCRIPTIONAL_REGULATION_BY_TP53
REACTOME_TRIGLYCERIDE_CATABOLISM
REACTOME_TRIGLYCERIDE_METABOLISM
REACTOME_VISUAL_PHOTOTRANSDUCTION
Effect Size:
1.0
0.5
0.0
-0.5
-1.0
Cases
Controls
White
Black
Adj. P
1
10^-1
10^-2
10^-3

Figure S2

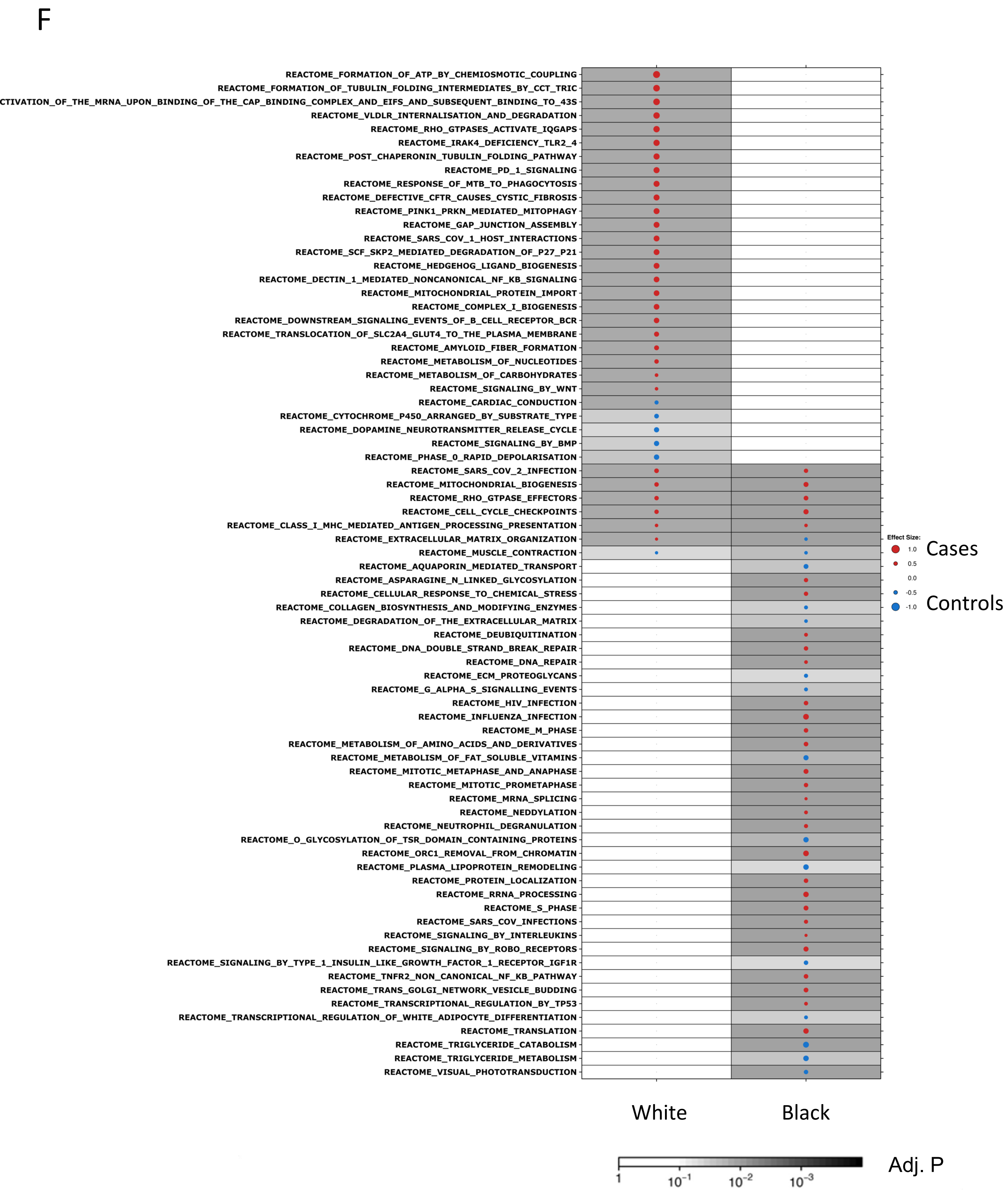
F
REACTOME_FORMATION_OF_ATP_BY_CHEMIOSMOTIC_COUPLING
REACTOME_FORMATION_OF_TUBULIN_FOLDING_INTERMEDIATES_BY_CCT_TRIC
CTIVATION_OF_THE_MRNA_UPON_BINDING_OF_THE_CAP_BINDING_COMPLEX_AND_EIFS_AND_SUBSEQUENT_BINDING_TO_43S
REACTOME_VLDLR_INTERNALISATION_AND_DEGRADATION
REACTOME_RHO_GTPASES_ACTIVATE_IQGAPS
REACTOME_IRAK4_DEFICIENCY_TLR2_4
REACTOME_POST_CHAPERONIN_TUBULIN_FOLDING_PATHWAY
REACTOME_PD_1_SIGNALING
REACTOME_RESPONSE_OF_MTB_TO_PHAGOCYTOSIS
REACTOME_DEFECTIVE_CFTR_CAUSES_CYSTIC_FIBROSIS
REACTOME_PINK1_PRKN_MEDIATED_MITOPHAGY
REACTOME_GAP_JUNCTION_ASSEMBLY
REACTOME_SARS_COV_1_HOST_INTERACTIONS
REACTOME_SCF_SKP2_MEDIATED_DEGRADATION_OF_P27_P21
REACTOME_HEDGEHOG_LIGAND_BIOGENESIS
REACTOME_DECTIN_1_MEDIATED_NONCANONICAL_NF_KB_SIGNALING
REACTOME_MITOCHONDRIAL_PROTEIN_IMPORT
REACTOME_COMPLEX_I_BIOGENESIS
REACTOME_DOWNSTREAM_SIGNALING_EVENTS_OF_B_CELL_RECEPTOR_BCR
REACTOME_TRANSLOCATION_OF_SLC2A4_GLUT4_TO_THE_PLASMA_MEMBRANE
REACTOME_AMYLOID_FIBER_FORMATION
REACTOME_METABOLISM_OF_NUCLEOTIDES
REACTOME_METABOLISM_OF_CARBOHYDRATES
REACTOME_SIGNALING_BY_WNT
REACTOME_CARDIAC_CONDUCTION
REACTOME_CYTOCHROME_P450_ARRANGED_BY_SUBSTRATE_TYPE
REACTOME_DOPAMINE_NEUROTRANSMITTER_RELEASE_CYCLE
REACTOME_SIGNALING_BY_BMP
REACTOME_PHASE_0_RAPID_DEPOLARISATION
REACTOME_SARS_COV_2_INFECTION
REACTOME_MITOCHONDRIAL_BIOGENESIS
REACTOME_RHO_GTPASE_EFFECTORS
REACTOME_CELL_CYCLE_CHECKPOINTS
REACTOME_CLASS_I_MHC_MEDIATED_ANTIGEN_PROCESSING_PRESENTATION
REACTOME_EXTRACELLULAR_MATRIX_ORGANIZATION
REACTOME_MUSCLE_CONTRACTION
REACTOME_AQUAPORIN_MEDIATED_TRANSPORT
REACTOME_ASPARAGINE_N_LINKED_GLYCOSYLATION
REACTOME_CELLULAR_RESPONSE_TO_CHEMICAL_STRESS
REACTOME_COLLAGEN_BIOSYNTHESIS_AND_MODIFYING_ENZYMES
REACTOME_DEGRADATION_OF_THE_EXTRACELLULAR_MATRIX
REACTOME_DEUBIQUITINATION
REACTOME_DNA_DOUBLE_STRAND_BREAK_REPAIR
REACTOME_DNA_REPAIR
REACTOME_ECM_PROTEOGLYCANS
REACTOME_G_ALPHA_S_SIGNALLING_EVENTS
REACTOME_HIV_INFECTION
REACTOME_INFLUENZA_INFECTION
REACTOME_M_PHASE
REACTOME_METABOLISM_OF_AMINO_ACIDS_AND_DERIVATIVES
REACTOME_METABOLISM_OF_FAT_SOLUBLE_VITAMINS
REACTOME_MITOTIC_METAPHASE_AND_ANAPHASE
REACTOME_MITOTIC_PROMETAPHASE
REACTOME_MRNA_SPLICING
REACTOME_NEDDYLATION
REACTOME_NEUTROPHIL_DEGRANULATION
REACTOME_O_GLYCOSYLATION_OF_TSR_DOMAIN_CONTAINING_PROTEINS
REACTOME_ORC1_REMOVAL_FROM_CHROMATIN
REACTOME_PLASMA_LIPOPROTEIN_REMODELING
REACTOME_PROTEIN_LOCALIZATION
REACTOME_RRNA_PROCESSING
REACTOME_S_PHASE
REACTOME_SARS_COV_INFECTIONS
REACTOME_SIGNALING_BY_INTERLEUKINS
REACTOME_SIGNALING_BY_ROBO_RECEPTORS
REACTOME_SIGNALING_BY_TYPE_1_INSULIN_LIKE_GROWTH_FACTOR_1_RECEPTOR_IGF1R
REACTOME_TNFR2_NON_CANONICAL_NF_KB_PATHWAY
REACTOME_TRANS_GOLGI_NETWORK_VESICLE_BUDDING
REACTOME_TRANSCRIPTIONAL_REGULATION_BY_TP53
REACTOME_TRANSCRIPTIONAL_REGULATION_OF_WHITE_ADIPOCYTE_DIFFERENTIATION
REACTOME_TRANSLATION
REACTOME_TRIGLYCERIDE_CATABOLISM
REACTOME_TRIGLYCERIDE_METABOLISM
REACTOME_VISUAL_PHOTOTRANSDUCTION
Effect Size:
1.0
Cases
0.5
0.0
-0.5
-1.0
Controls
White
Black
1
10^-1
10^-2
10^-3
Adj. P

Figure S2

G

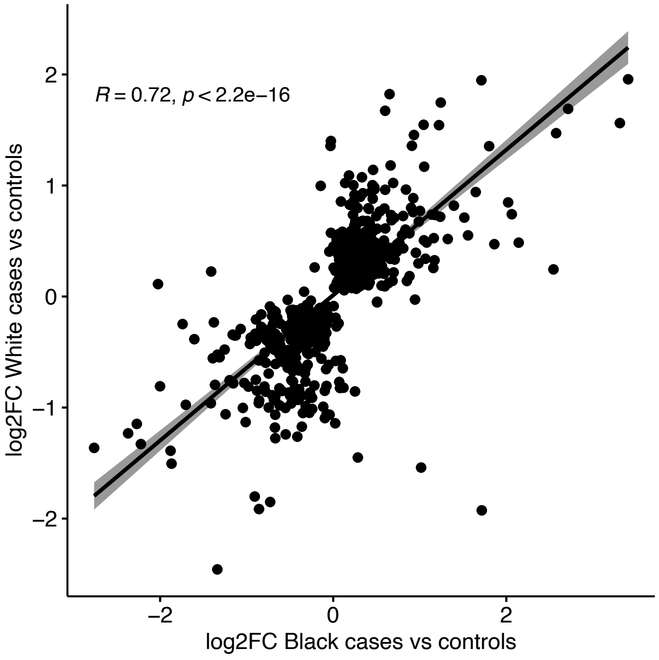

Figure S2: Differential gene expression by SRR and outcome groups

A) GSEA Hallmark analysis of differentially expressed genes between DCIS from White and Black women. B) Volcano plot of differentially expressed genes from Black case-vs-controls analysis. Genes with adj. P<0.05 in red (n=266). C) Volcano plot of differentially expressed genes from White case-vs-controls analysis. Genes with adj. P<0.05 in red (n=812). D – F) GSEA of differentially expressed genes between DCIS from White cases vs controls (left column) and Black cases vs controls (right column), respectively, using GO terms (D), KEGG (E), and Reactome (F) gene sets. A, D, E, F) Dot size and color represent the magnitude and direction of pathway deregulation. Background shading indicates FDR. Effect size and FDR from GSEA algorithm. G) Scatter plot of log2FC values of the 812 genes included in the HTAN DCIS classifier from Black case-vs-control analysis (x-axis) versus White case-vs-control analysis (y-axis). Correlation coefficient and P-value from Pearson correlation test.

Figure S3

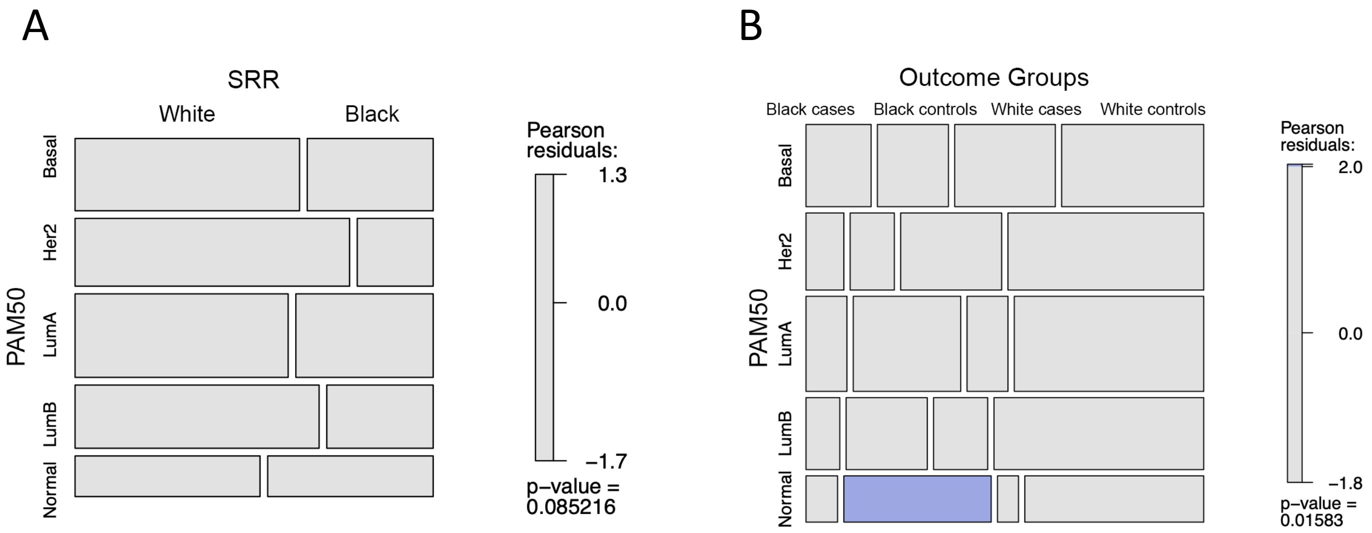

Figure S3: PAM50 by SRR and outcome groups

Mosaic plots showing distribution of PAM50 by SRR (A) and SRR and outcome groups (B). P-values from Chi^2 test.

Figure S4

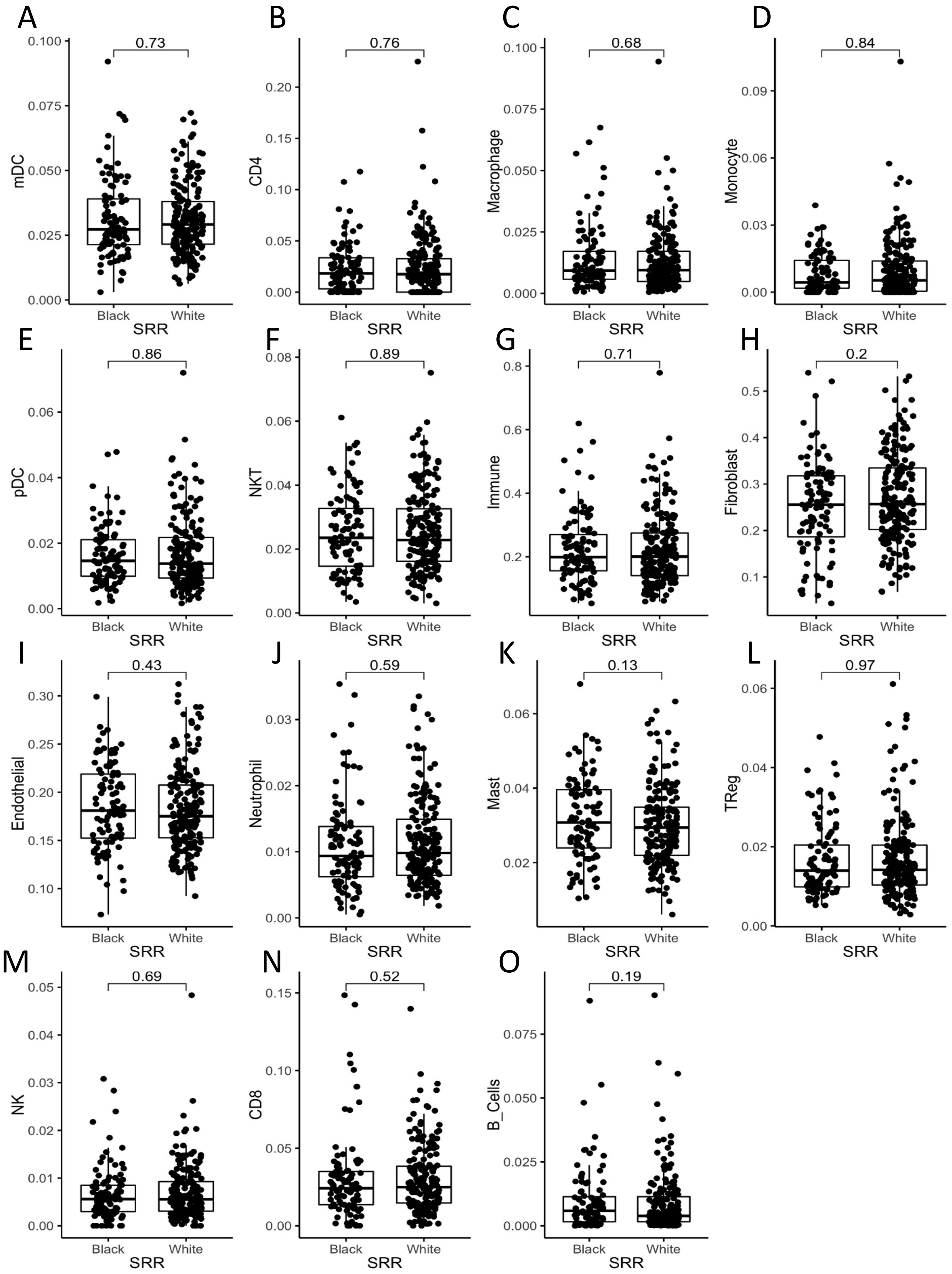

A
B
C
D
E
F
G
H
I
J
K
L
M
N
O
mDC
CD4
Macrophage
Monocyte
pDC
NKT
Immune
Fibroblast
Endothelial
Neutrophil
Mast
TReg
NK
CD8
B_Cells
0.73
0.76
0.68
0.84
0.86
0.89
0.71
0.2
0.43
0.59
0.13
0.97
0.69
0.52
0.19
Black
White
SRR

Figure S4: Cell type distribution by SRR.

A – O) Inferred cell type distribution from RNA-seq data using CibersortX. Boxplot represents median, 0.25 and 0.75 quantiles with whiskers at 1.5x interquartile range. P-values from Wilcoxon rank-sum test. mDC = Myeloid dendritic cells. pDC = Plasmacytoid dendritic cells.

Figure S5

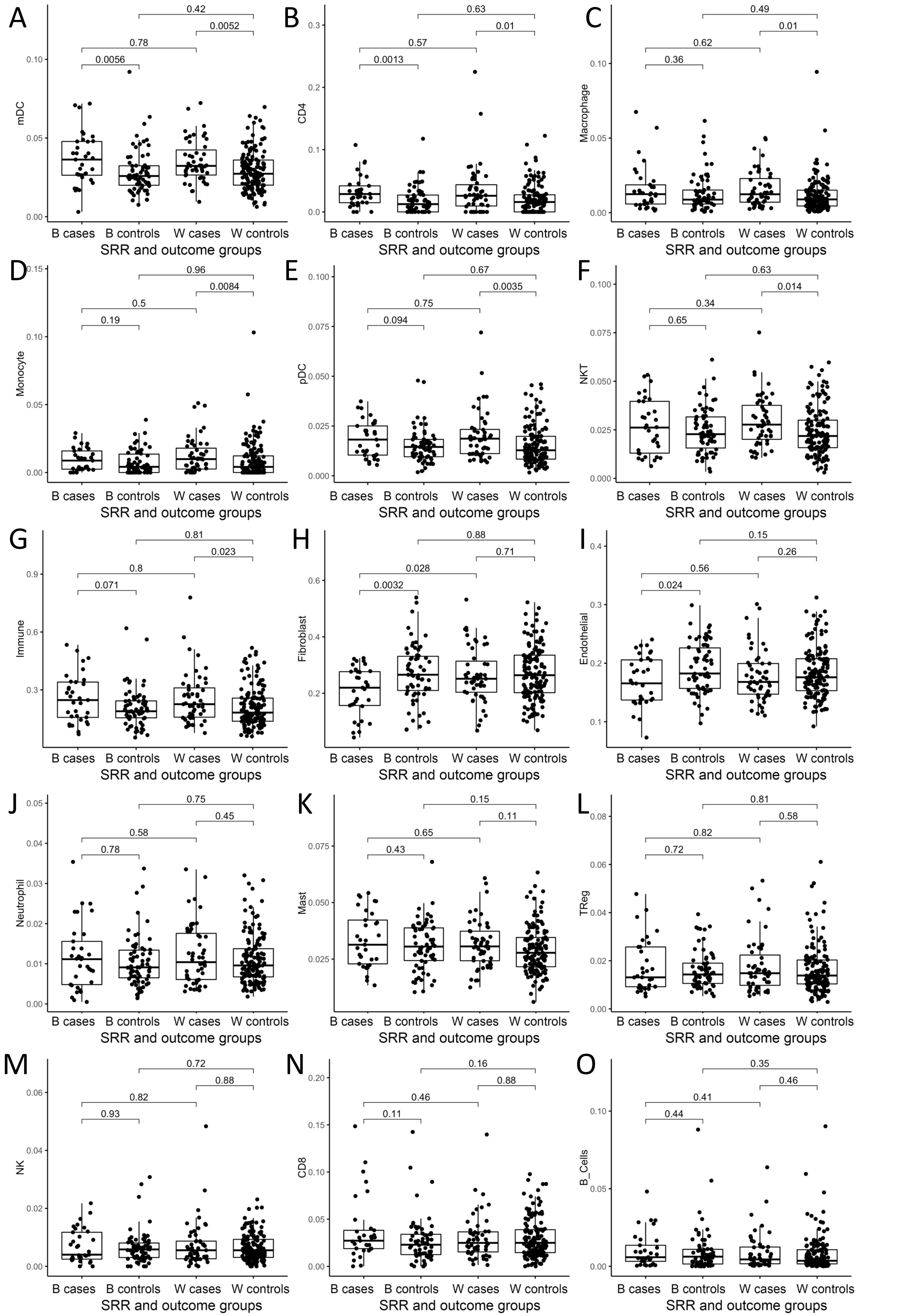

A
mDC
0.00
0.05
0.10
0.0056
0.78
0.0052
0.42
B cases
B controls
W cases
W controls
SRR and outcome groups
B
CD4
0.0
0.1
0.2
0.3
0.0013
0.57
0.01
0.63
B cases
B controls
W cases
W controls
SRR and outcome groups
C
Macrophage
0.00
0.05
0.10
0.36
0.62
0.01
0.49
B cases
B controls
W cases
W controls
SRR and outcome groups
D
Monocyte
0.00
0.05
0.10
0.15
0.19
0.5
0.0084
0.96
B cases
B controls
W cases
W controls
SRR and outcome groups
E
pDC
0.000
0.025
0.050
0.075
0.100
0.094
0.75
0.0035
0.67
B cases
B controls
W cases
W controls
SRR and outcome groups
F
NKT
0.000
0.025
0.050
0.075
0.100
0.65
0.34
0.014
0.63
B cases
B controls
W cases
W controls
SRR and outcome groups
G
Immune
0.3
0.6
0.9
0.071
0.8
0.023
0.81
B cases
B controls
W cases
W controls
SRR and outcome groups
H
Fibroblast
0.2
0.4
0.6
0.0032
0.028
0.71
0.88
B cases
B controls
W cases
W controls
SRR and outcome groups
I
Endothelial
0.1
0.2
0.3
0.4
0.024
0.56
0.26
0.15
B cases
B controls
W cases
W controls
SRR and outcome groups
J
Neutrophil
0.00
0.01
0.02
0.03
0.04
0.05
0.78
0.58
0.45
0.75
B cases
B controls
W cases
W controls
SRR and outcome groups
K
Mast
0.025
0.050
0.075
0.43
0.65
0.11
0.15
B cases
B controls
W cases
W controls
SRR and outcome groups
L
TReg
0.00
0.02
0.04
0.06
0.08
0.72
0.82
0.58
0.81
B cases
B controls
W cases
W controls
SRR and outcome groups
M
NK
0.00
0.02
0.04
0.06
0.93
0.82
0.88
0.72
B cases
B controls
W cases
W controls
SRR and outcome groups
N
CD8
0.00
0.05
0.10
0.15
0.20
0.11
0.46
0.88
0.16
B cases
B controls
W cases
W controls
SRR and outcome groups
O
B_Cells
0.00
0.05
0.10
0.44
0.41
0.46
0.35
B cases
B controls
W cases
W controls
SRR and outcome groups

Figure S5

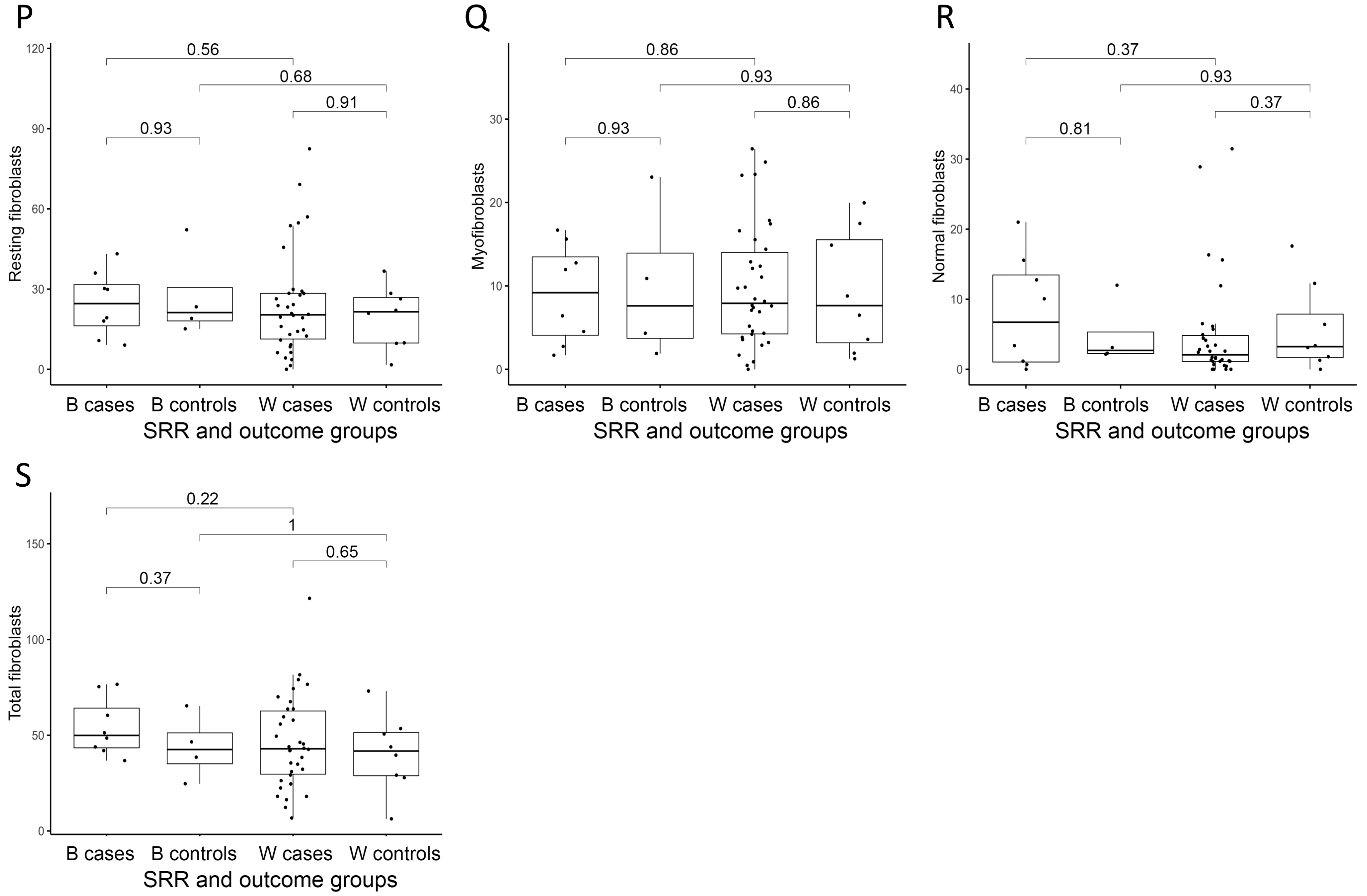

Figure S5: Cell type distribution by SRR and outcome groups

A – O) Inferred cell type distribution from RNA-seq data using CibersortX. mDC = Myeloid dendritic cells. pDC = Plasmacytoid dendritic cells. P – S) Fibroblast phenotypes by SRR and outcome groups in MIBI sample-level data (n=54). P) Resting fibroblasts. Q) Myofibroblasts. R) Normal fibroblasts. S) Total fibroblasts. A – S) Boxplot represents median, 0.25 and 0.75 quantiles with whiskers at 1.5x interquartile range. P-values from Wilcoxon rank-sum test. B: Black. W: White.

Figure S6

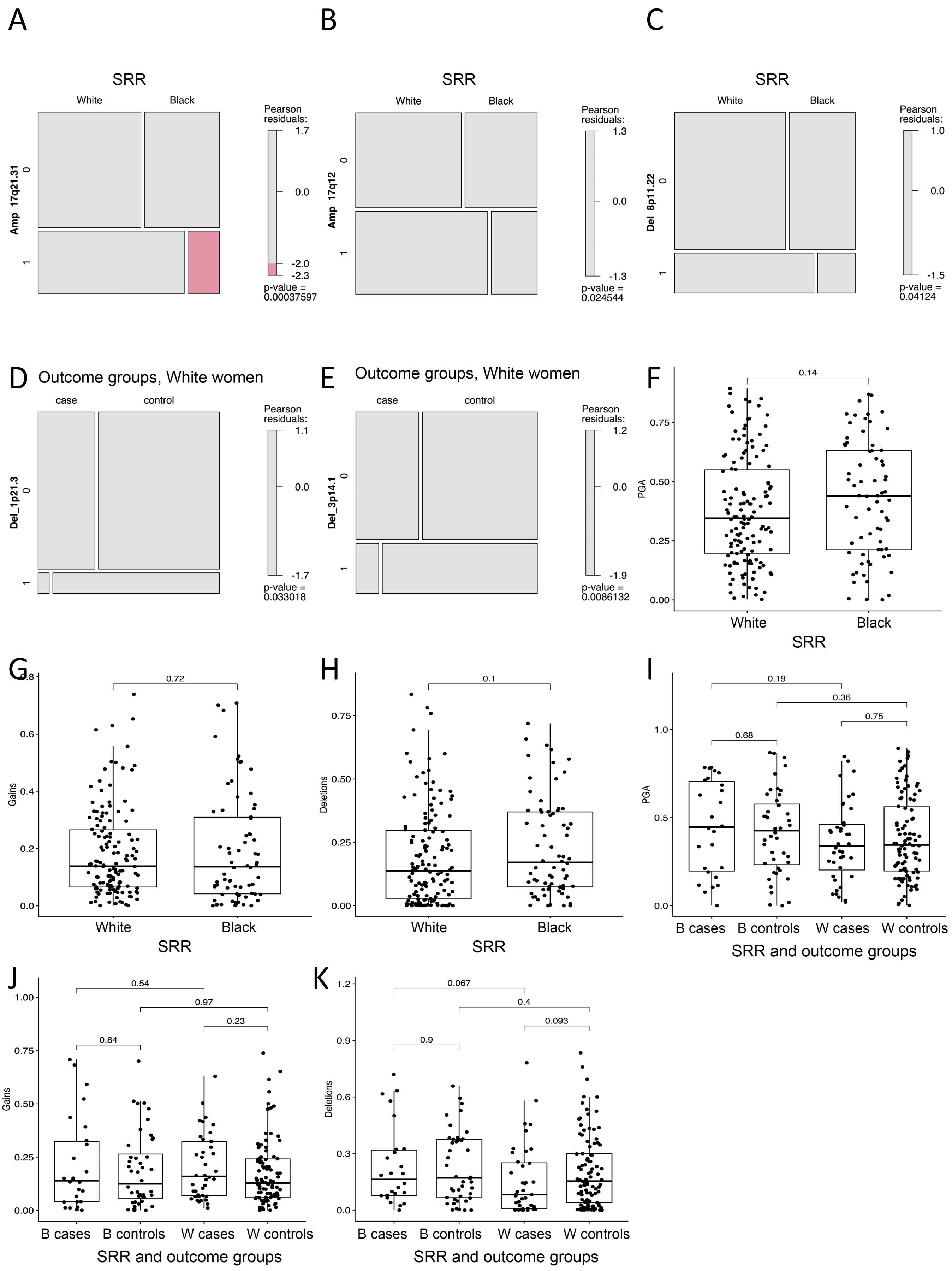
A
SRR
White
Black
Amp 17q21.31
0
1
Pearson residuals:
1.7
0.0
-2.0
-2.3
p-value = 0.00037597
B
SRR
White
Black
Amp 17q12
0
1
Pearson residuals:
1.3
0.0
-1.3
p-value = 0.024544
C
SRR
White
Black
Del 8p11.22
0
1
Pearson residuals:
1.0
0.0
-1.5
p-value = 0.04124
D
Outcome groups, White women
case
control
Del_1p21.3
0
1
Pearson residuals:
1.1
0.0
-1.7
p-value = 0.033018
E
Outcome groups, White women
case
control
Del_3p14.1
0
1
Pearson residuals:
1.2
0.0
-1.9
p-value = 0.0086132
F
0.14
PGA
0.75
0.50
0.25
0.00
White
Black
SRR
G
0.72
Gains
0.6
0.4
0.2
0.0
White
Black
SRR
H
0.1
Deletions
0.75
0.50
0.25
0.00
White
Black
SRR
I
0.19
0.36
0.75
0.68
PGA
1.0
0.5
0.0
B cases
B controls
W cases
W controls
SRR and outcome groups
J
0.54
0.97
0.23
0.84
Gains
1.00
0.75
0.50
0.25
0.00
B cases
B controls
W cases
W controls
SRR and outcome groups
K
0.067
0.4
0.093
0.9
Deletions
1.2
0.9
0.6
0.3
0.0
B cases
B controls
W cases
W controls
SRR and outcome groups

Figure S6: Genomic alterations by SRR and outcome groups

A – C) Mosaic plots showing distribution of significant CNVs by SRR. D – E) Mosaic plots showing distribution of significant CNVs by 5-year outcome groups in White women only. A – E) P-values from Chi^2 test. F – H) Boxplots showing Proportion of the Genome copy number Altered (PGA, F), Gains (G), and Deletions (H) by SRR. I - K) Boxplots showing PGA (I), Gains (J), and Deletions (K) by SRR and outcome groups. F – K) Boxplots represent median, 0.25 and 0.75 quantiles with whiskers at 1.5x interquartile range. P-values from Wilcoxon rank-sum test.
